# Microfluidic-Assisted Preparation of Targeted pH-Responsive Polymeric Micelles Improves Gemcitabine Effectiveness in PDAC: In Vitro Insights

**DOI:** 10.3390/cancers14010005

**Published:** 2021-12-21

**Authors:** Rosa Maria Iacobazzi, Ilaria Arduino, Roberta Di Fonte, Angela Assunta Lopedota, Simona Serratì, Giuseppe Racaniello, Viviana Bruno, Valentino Laquintana, Byung-Chul Lee, Nicola Silvestris, Francesco Leonetti, Nunzio Denora, Letizia Porcelli, Amalia Azzariti

**Affiliations:** 1 Laboratory of Experimental Pharmacology, IRCCS Istituto Tumori “Giovanni Paolo II”, 70124 Bari, Italy; r.m.iacobazzi@oncologico.bari.it (R.M.I.); r.difonte@oncologico.bari.it (R.D.F.); vivibr.fismat@gmail.com (V.B.); a.azzariti@oncologico.bari.it (A.A.); 2 Department of Pharmacy–Pharmaceutical Sciences, University of Bari, 70125 Bari, Italy; ilaria.arduino@uniba.it (I.A.); angelaassunta.lopedota@uniba.it (A.A.L.); giuseppe.racaniello@uniba.it (G.R.); valentino.laquintana@uniba.it (V.L.); francesco.leonetti@uniba.it (F.L.); 3 Laboratory of Nanotechnology, IRCCS Istituto Tumori “Giovanni Paolo II”, 70124 Bari, Italy; s.serrati@oncologico.bari.it; 4 Department of Nuclear Medicine, Seoul National University Bundang Hospital, Seoul National University College of Medicine, Seongnam 13620, Korea; leebc@snu.ac.kr; 5 Medical Oncology Unit, IRCCS Istituto Tumori “Giovanni Paolo II” of Bari, 70124 Bari, Italy; n.silvestris@oncologico.bari.it; 6 Department of Biomedical Sciences Human Oncology, University of Bari “Aldo Moro”, 70124 Bari, Italy

**Keywords:** pancreatic ductal adenocarcinoma, tumor microenvironment, drug resistance, drug delivery, controlled release, uPAR, active drug targeting, pH-responsiveness

## Abstract

**Simple Summary:**

This research suggests a new potential therapeutic approach to pancreatic ductal adenocarcinoma to improve drug effectiveness and overcome drug resistance. A double actively targeted gemcitabine delivery system, consisting of polymeric micelles, was developed by microfluidic technique to ensure a narrow size distribution, a good colloidal stability, and drug-encapsulation efficiency for the selective and controlled release of the loaded drug, in response to the pH variations and uPAR expression in tumors. In vitro studies assessed that the release of the drug in the acidic environment was higher than in the neutral one, and that the pH-responsive and uPAR-targeted polymeric micelles enhanced the antitumor properties of gemcitabine in models resembling the pancreatic tumor microenvironment.

**Abstract:**

Pancreatic ductal adenocarcinoma (PDAC) represents a great challenge to the successful delivery of the anticancer drugs. The intrinsic characteristics of the PDAC microenvironment and drugs resistance make it suitable for therapeutic approaches with stimulus-responsive drug delivery systems (DDSs), such as pH, within the tumor microenvironment (TME). Moreover, the high expression of uPAR in PDAC can be exploited for a drug receptor-mediated active targeting strategy. Here, a pH-responsive and uPAR-targeted Gemcitabine (Gem) DDS, consisting of polymeric micelles (Gem@TpHResMic), was formulated by microfluidic technique to obtain a preparation characterized by a narrow size distribution, good colloidal stability, and high drug-encapsulation efficiency (EE%). The Gem@TpHResMic was able to perform a controlled Gem release in an acidic environment and to selectively target uPAR-expressing tumor cells. The Gem@TpHResMic displayed relevant cellular internalization and greater antitumor properties than free Gem in 2D and 3D models of pancreatic cancer, by generating massive damage to DNA, in terms of H2AX phosphorylation and apoptosis induction. Further investigation into the physiological model of PDAC, obtained by a co-culture of tumor spheroids and cancer-associated fibroblast (CAF), highlighted that the micellar system enhanced the antitumor potential of Gem, and was demonstrated to overcome the TME-dependent drug resistance. In vivo investigation is warranted to consider this new DDS as a new approach to overcome drug resistance in PDAC.

## 1. Introduction

The PDAC represents a very aggressive type of tumor, which is not easy to diagnose. According to the national statistics, in Italy it is a cancer with poor prognosis, with a 5-year survival rate of 8%, while the 10-year survival rate is 3% [1]. These statistics are similar to the ones from American Cancer Society, with a 5-year survival rate of 10.1% [2]. Cancer tissues show significant structural changes compared with healthy tissues. The turning point from the pathophysiological point of view is represented by the desmoplasia of the stroma, which is marked by a dramatic increase in the proliferation of cancer-associated fibroblasts (CAF) and increased deposition of many extracellular matrix components [3], that deforms the normal architecture of pancreatic tissues [3,4,5]. The external stroma, that is close enough to the blood circulation where oxygen is sufficient for oxidative phosphorylation, shows a pH value quite similar to the physiological value, of almost 7.4. Conversely, the tumor core is away from the blood vessel, so there is a predominance of anaerobic glycolysis, which leads to a lower pH of almost 6.8, due to the high production of acidic products, such as lactic acid (Warburg effect [6]). In addition, poor lymphatic drainage contributes to the acidity of the tumor microenvironment [7]. All these aspects make pancreatic cancer less sensitive to conventional treatments, including chemotherapy and radiotherapy. Currently, Gemcitabine/Nabpaclitaxel (Gem/NAB), FOLFIRINOX (Oxaliplatin, Leucovorin, Irinotecan, and 5-Fluorouracil), Gemcitabine (Gem), and Capecitabine are the most successful chemotherapy regimens for PDAC [1], depending on patients’ condition [8]. Although the FOLFIRINOX regimen has been confirmed to be superior to Gem for the treatment of PDAC, the higher toxicity of such a treatment limits its application [9]. For this reason, monotherapy or combined therapy with Gem is still the golden standard of current PDAC chemotherapy regimens, even though there is evidence that PDAC cells participate in the pathogenesis of Gem resistance [4,10,11]. To overcome resistance, higher doses of Gem are required, which lead to severe systemic toxicity [12,13]. The control of the drug release site and time could improve drug efficacy, with lower doses and toxicity [14,15].

The intrinsic characteristics of the PDAC microenvironment and drug resistance make it suited to therapeutical approaches with stimulus-responsive drug delivery systems (DDSs), such as pH, within the tumor microenvironment. Through such a DDS, it is possible to selectively release the drug in the acidic tumor microenvironment while maintaining the drug’s stability within the DDSs in normal tissue [16]. Several examples of pH-responsive DDSs have been proposed over time, including chitosan-coated mesoporous silica nanoparticles, liposomes, polymeric micelles, and nanospheres [5,14,15,17,18,19,20,21].

The distribution of drug-loaded nanoparticles in the body is determined by their dimensions and surface properties, and also by the enhanced permeability and retention (EPR) effect, which is the rational basis for passive targeting in antitumor drug delivery [22]. The outer shell of nanoparticles is usually able to control biodistribution, drug release, and excretion, while the inner core is responsible for loading the capacity and stability of the drugs [8,23]. In this perspective, we focused on the development of pH-responsive polymeric micelles with average diameters of <200 nm, and we used a microfluidic-assisted preparation of such a nano-system, in order to address a controlled and high batch-to-batch reproducibility [24] and a greater control of nanoparticles properties, including the drug encapsulation efficiency and the monodispersity of batches [25,26,27,28]. The nano-system consisted of a mixture of three di-block copolymers, namely the poly(lactic-co-glycolic acid)-polyethylene glycol (PLGA-PEG), to confer stealth properties, and the poly(lactic-co-glycolic acid)-poly-L-lysine conjugated to the 2,3-dimethylmaleic anhydride (PLGA-PLL-DMA) as a pH-responsive moiety. In addition, to improve specific targeting to the PDAC, the surface of such micelles was properly functionalized with a selective ligand for uPAR receptor, which is highly expressed in PDAC cells [12,13], such as the peptide AE105 [5]. The microfluidic technique has already been successfully employed to control the size, shape, and polydispersity of polymeric micelles [29,30,31,32,33,34,35,36]; however, to the best of our knowledge, there have been few reports on microfluidics-based pH-responsive polymeric micellar delivery systems [37,38,39] and no one has applied these to PDAC cancer research. In particular, Feng et al. [37] obtained the pH-responsive micelles by mixing two different block copolymers (the PLGA and the poly(ethylene glycol)-poly(2-(diisopropylamino)ethyl methacrylate(PEG-b-PDPA)); additionally, Albuquerque et al. [38], using the poly([N-(2-hydroxypropyl)]methacrylamide)-bpoly [2-(diisopropylamino)ethyl methacrylate] (PHPMAm-b-PDPAn), blocked copolymers, thus obtaining quasi-monodisperse assemblies, characterized by more consistent biodistribution and cellular uptake. In our work, with the aid of the microfluidic technique, we were able to develop pH-responsive polymeric micelles, simultaneously targeted to a specific receptor (uPAR), by mixing three different di-block copolymers (PLGA-PEG, PLGA-PLL-DMA, and PLGA-PEG-AE105), mentioned above, while still obtaining a monodisperse and therefore optimal formulation for a potential therapeutic use in PDAC.

Gem was chosen as a model drug to load into the micellar system, in order to demonstrate that the newly synthesized nano-system improved drug efficacy in both 2D and 3D models of pancreatic cancer and, in a more physiologically relevant system, which was obtained from co-culture of pancreatic cancer-associated fibroblasts (CAF) and pancreatic cancer cells in 3D.

## 2. Materials and Methods

Chemical reagents and organic solvents were of analytical grade and used as obtained without further purification. Poly (ethylene glycol) BioUltra 4000 (PEG), Resomer^®^ RG 502 H, Poly (D, L-lactide-co-glycolide) acid terminated (PLGA), MW 7000−17000, Poly-L-lysine hydrobromide (PLL), molecular weight 15000–30000, 2.3-dimethylmaleic anhydride (DMA), N, N′-dicyclohexylcarbodiimide (DCC), N-Hydroxy-succinimide (NHS), Triethylamine (TEA), N, N-Dimethylformamide anhydrous ≥99.9% (DMF), tetrahydrofuran (THF), dichloromethane (DCM), Amicon tubes ultra−15 for centrifuge 3000× *g* Da, and PBS (phosphate buffered solution) were purchased from Sigma-Aldrich (Merk Life Science S.r.l., Milano Italy). BODIPY 650/665 alkyne dye was purchased from Lumi Probe. Gemcitabine was purchased from Selleckchem (Munich, Germany) and the NH_2_-PEG-AE105 peptide was provided by ChinaPeptides Co., Ltd. (Shanghai, China).

### 2.1. Synthesis of Copolymers

The synthesis and characterization of the activated PLGA polymer, and of PLGA-PEG, PLGA-PLL, PLGA-PLL-DMA, and PLGA-PEG-AE105 copolymers are reported in the Supplementary S2. Materials and Methods.

### 2.2. Determination of Critical Micelle Concentration (CMC)

To assess the CMC values of PLGA-PEG, PLGA-PLL-DMA, and of the mixture PLGA-PEG/PLGA-PLL-DMA, the protocol described in the Malvern application note was used [40]. Kcps values obtained with DLS analysis of the copolymers suspensions were evaluated in deionized water as a function of concentration values [41].

### 2.3. Preparation and Characterization of the Micelles

#### 2.3.1. Preparation of Glass Capillary Microfluidic Chip

The microfluidic platform was constructed by mounting borosilicate glass capillaries on a glass rods, as previously explained [25]. One end of the cylindrical glass capillary (World Precision Instruments, Inc., Sarasota, FL, USA), with inner and outer diameters of 580 and 1000 µm, respectively, was tapered using a micropipette puller (DMZ—Universal Puller) to a diameter of 20 μm; this diameter was further enlarged to approximately 80 µm using sandpaper (Dexter, RONCHIN, France). This cylindrical tapered capillary was inserted and coaxially aligned into the left end of the cylindrical capillary with an inner dimension of 1100 µm. For the production of the micelles, two miscible liquids, one forming the inner and the other the outer phase, were pumped independently into the microfluidic device with a regular flow through the use of a syringe connected to polypropylene tubes. The flow rate of the different liquids was controlled by pumps (Kd Scientific, Holliston, MA, USA).

#### 2.3.2. Preparation of Micelles by Microfluidic Technique

**Figure 1:**
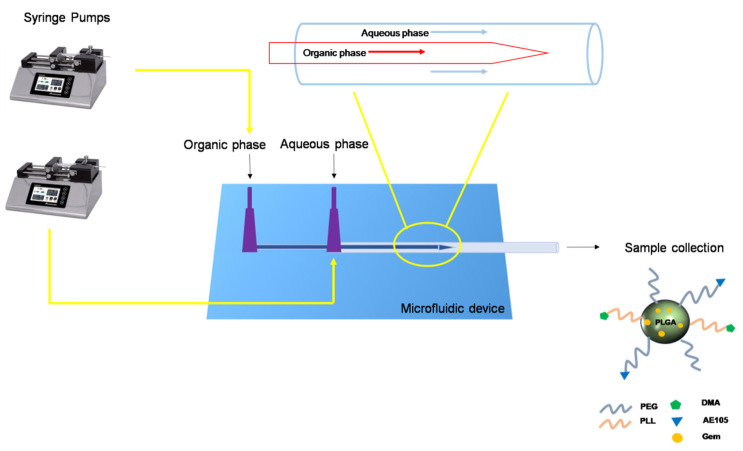
Schematic representation of the microfluidic platform for the production of micelles.

The non-responsive, pH-responsive, and the targeted pH-responsive micelles (NoResMic, pHResMic, and TpHResMic, respectively, Table 1), were prepared by co-flow nanoprecipitation in a glass capillary microfluidics device (Figure 1). The organic phase consisted of a mixture of 5 mg/mL of PLGA-PEG and 5 mg/mL PLGA-PLL (-DMA) in DMF (for the empty NoResMic and pHResMic) and 0.75 mg/mL of Gem (for Gem@pHResMic), while for the preparation of micelles, functionalized on the surface with AE105 peptide (TpHResMic and Gem@TpHResMic), the organic phase was composed of 4 mg/mL of PLGA-PEG, 5 mg/mL PLGA-PLL-DMA, 1 mg of PLGA-PEG-AE105, and 0.75 mg/mL of Gem (only for Gem@TpHResMic). In all formulations, the aqueous phase consisted of 1% (*w*/*v*) of Pluronic F68 in basic water pH 8.8.

The organic and aqueous phases corresponded to the inner and outer fluids, respectively. With the usage of two microfluidics pumps, we set the flow rate of the inner (500 µL/min) and the outer solution (1 mL/min) and we kept the flow-rate constant.

The liquids flowed from their respective syringes into the devices through tiny tubes.

After preparation, the suspension was stirred for 30 min. To remove the surfactant and non-encapsulated drug, the produced micelles were carefully purified using centrifugal concentrators (Centrifugal Filter Units-Amicon Ultra 50kDa) by PBS 1× pH 8.8 at 4 °C, 3500 rpm, for 5 min (5 times). After purification, the micelles were filtered with a 0.45 nm nylon filter and finally they were stored at 4 °C.

To determine the cellular uptake of micelles, BODIPY (BDP) was added to the organic phase (1/100 *w*/*w* ratio of BDP/copolymers).

#### 2.3.3. Particle Size, Size Distribution, and Surface Charge

After their preparation, the micelles were characterized by means of dynamic light scattering to obtain information about their size, polydispersity index (PDI), and zeta (ζ)-potential, using a Malvern Zetasizer Nano instrument (Malvern Ltd., Malvern, UK). Details are reported the Supplementary S2. Materials and Methods.

### 2.4. Stability Studies

In order to estimate the micelles short-term stability, the size distribution of pHResMic and TpHResMic was measured in PBS (pH 7.4). Details are reported in the Supplementary S2. Materials and Methods.

### 2.5. Evaluation of Drug Encapsulation Efficiency

The encapsulation efficiency (EE%) values of GEM loaded in micelles were determined as reported in the Supplementary S2. Materials and Methods.

### 2.6. In Vitro Drug Release Study

Studies of Gem release from TpHResMic were performed using Franz cells [25], and experiments were carried out at two different pH (6.8 and 7.4). Details are reported in the Supplementary S2. Materials and Methods.

### 2.7. Cell Culture

Human PDAC PANC−1 cells (ATCC-CRL−1469), human pancreas adenocarcinoma ascites metastasis cells AsPC−1 (ATCC-CRL−1682), and undifferentiated human pancreatic carcinoma MIAPaCa-2 cells (ATCC-CRL−1420) were purchased from ATCC (LGC Standard s.r.l., Sesto San Giovanni (MI), Italy), and were cultured as previously reported [42]. Human pancreatic cancer stllate cancer-associated fibroblasts (CAF08, PC00B5) were purchased from Vitro Biopharma (Golden, Colorado). All materials for cell culturing were purchased from EuroClone (Pero, Milan, Italy).

For PANC−1 tumor homo-spheroids formation, cells were seeded at a density of 5000 cells per well on 96-well Corning Spheroid Microplate (Corning, NY, USA) and incubated at 37 °C under 5% CO_2_ for 5 days before performing experiments. The spheroids of PANC−1 in co-culture with CAF cells were obtained, as described above for homospheroids using a 2:1 PANC−1:CAF ratio. Spheroid formation was assessed using OLYMPUS CKX41 microscope (OLYMPUS, Tokyo, Japan).

### 2.8. uPAR Expression Evaluation by Flow Cytometry (FCM) Analysis

To verify the expression of uPAR receptors on PDAC cells, flow cytometry (FCM) analysis was performed. Cells were seeded in 60 mm dishes at a density of 500,000/well and incubated for 1 day at 37 °C. Afterward, the cells were harvested, washed twice, resuspended in ice-cold PBS without Ca^2+^ and Mg^2+^, and incubated to labelling with antihuman PerCP-eFluor 710-CD87 (uPAR) antibody (eBioscience^TM^, Invitrogen, Thermo Fisher Scientific, Waltham, MA, USA) for 30 min at 2–8 °C in the dark. After staining, cells were washed with PBS without Ca^2+^ and Mg^2+^ and analyzed using an Attune NxT Acoustic Focusing Cytometer (Thermo Fisher Scientific, Waltham, MA, USA) equipped with four lasers (405 nm (violet), 488 nm (blue), 561 nm (yellow), and 637 nm (red)) for sample reading. The final data were analyzed using Attune NxT Analysis Software (Thermo Fisher Scientific, Waltham, MA, USA).

### 2.9. In Vitro Uptake Study on 2D and 3D Model of PDAC Cells

To assess the pH dependent internalization of BDP-loaded, pH-responsive micelles (BDP@pHResMic), the uptake studies were conducted in vitro in 2D models of PDAC at two different pH and temperature conditions, pH 6.8 or pH 7.4 and 37 °C or 4 °C, by FCM analysis.

The receptor-mediated uptake ability of BDP-loaded, uPAR-targeted, and pH-responsive micelles (BDP@TpHResMic) was assessed in pH 6.8 medium, at 37 °C and 4 °C, in 2D and 3D models of PDAC cell lines, by FCM and fluorescence imaging, respectively.

In detail, the uptake experiment in 2D was conducted as described in Iacobazzi et al. [43]. PANC−1, AsPC−1, and MIAPaCa-2 cells were seeded in 6-well plates (Corning, NY, USA) at a density of 300,000 cells per well and, after attachment, incubated with BDP@micelles (at a concentration of 0.1 μM, in terms of the loaded fluorescent dye) for 1 h at 37 °C and at 4 °C (for external binding detection). To determine external binding, the cells were preventively cooled on ice for 25 min before starting the experiment and were maintained at 4 °C for the rest of the experiment. After the incubation, the cells were detached with trypsin-EDTA, washed with cold PBS, and finally re-suspended in PBS without Ca^2+^ and Mg^2+^. Both the external-binding fluorescence and the cell-internalized fluorescence were measured by the Attune NxT Acoustic Focusing Cytometer (Thermo Fisher Scientific, Waltham, MA, USA). A total 10,000 events were counted in the viable gate and the geometric mean of the viable cell population exposed to BDP@micelles was used to determine their internalization, after correction for cell and for micelles auto-fluorescence. The amount of internalized micelles was calculated by subtracting the 4 °C values from the 37 °C values.

The competition study was performed on AsPC−1 cells to confirm the ability of BDP@TpHResMic to bind the uPAR receptor. After 20 min of pre-incubation with the AE105 peptide at a concentration of 10 μM, the cells were co-incubated for 1 h at 37 °C and then processed, as described above, to quantify the amount of internalized fluorescence.

Data were interpreted using the Attune NxT Analysis Software (Thermo Fisher Scientific, Waltham, MA, USA) and represent mean ± SD, *n* = 3.

For the qualitative cell internalization analysis in a 3D PDAC model, PANC−1 spheroids were generated from 5,000 cells in 5 days, utilizing 96-well Corning^®^ Spheroid Microplates. Then, spheroids were incubated for 2 h with targeted pH-responsive micelles and with non-targeted pH-responsive micelles at a concentration of 0.1 μM, in terms of the loaded BDP. For nucleic acid staining spheroids were incubated for 30 min with Hoechst 33342 dye (2 μg/mL, InvitrogenTM, Eugene, Oregon, USA). Finally, spheroids were washed and recovered with PBS without Ca^2+^ and Mg^2+^ and visualized utilizing the Celldiscoverer 7 microscope (Carl Zeiss Microscopy, Oberkochen, Germany), with a Plan-Apochromat 5×/0.35 objective and optavar 0.5× tube lens.

### 2.10. In Vitro Proliferation Study

PANC−1, AsPC−1, and MIAPaCa-2 cell lines were seeded at a density of 5,000 cells/well for 24 h in 96-well plates (Corning, NY, USA). Subsequently, the culture medium was replaced with 100 µL of fresh medium adjusted for pH value at 7.4 or 6.8 and containing dilutions of Gem@pHResMic, Gem@TpHResMic, empty micelles, and Gem alone as the reference compounds. The cells were treated with the tested compounds at 37 °C for 24 h, then washed out and cultured in a fresh culture medium, at pH 7.4 or 6.8, for further 48 h. The tested concentration range was 0.01-50 µM for Gem, both loaded or not, and 0.3−1500 µg/mL for empty micelles. Cell viability was assessed using MTT assay as previously described [43], and results were expressed as dose/effect plots of the mean of three different experiments at each tested dose or IC_50_ values obtained using nonlinear regression in CalcuSyn v.1.1.1 software (Biosoft, Cambridge, UK).

The antiproliferative effect of Gem@TpHResMic and of free Gem was also evaluated in 3D models of PANC−1 cells and in tumor spheroids in co-culture with CAF cells (ratio 2:1). After incubation with Gem@TpHResMic or free Gem we recovered the spheroids and disaggregated them with TrypLE^TM^ Express solution (GIBCO, Thermo Fisher Scientific). Afterward, we evaluated the percentage of died cells by FCM using the Fixable Viability Dye eFluor™ 780 (Thermo Fisher Scientific, Waltham, MA USA), which labels dead cells. In addition, the same spheroids were stained with the Ready probes^TM^ cell viability imaging kit (blue/red) (Invitrogen, Thermo Fisher Scientific, Eugene, OR, USA), while the CAF were stained by using PKH67-green dye (Sigma Aldrich, Merck Life Science S.r.l., Milano, Italy), according to instruction provided by the manufacturers. The spheroids were visualized utilizing the Celldiscoverer 7 microscope (Carl Zeiss Microscopy), with a Plan-Apochromat 5×/0.35 objective and optavar 0.5× tube lens.

### 2.11. Evaluation of the Cell Cycle Progression, Apoptosis Induction and γH_2_AX Analysis

#### 2.11.1. Cell Cycle Analysis

The human pancreatic cancer cells were seeded in 60 mm dishes at a density of 300,000/well and incubated at pH 6.8 and for 24 h, or 24 h followed by 48 h, washing out with Gem@TpHResMic or free Gem. Gem was used at 3 µM, 5 µM, and 54 nM in PANC−1, MIAPaCa-2, and AsPC−1 cells, respectively. Then, the cells were harvested, washed twice in ice-cold PBS pH 7.4, fixed in 4 mL of 70% ethanol, and stored at −20 °C until analysis. The cell cycle modulation induced by treatments was studied, as previously described [42], by propidium iodide (PI) staining; the pellet was resuspended in PBS without Ca^2+^ and Mg^2+^, containing 1 mg/mL RNase, 0.01% NP40, and 20 µg/mL PI (Sigma); then, FCM analysis was performed by Attune NxT Acoustic Focusing Cytometer (Thermo Fisher Scientific, Waltham, MA, USA). Data were interpreted using the Attune NxT Analysis Software (Thermo Fisher Scientific, Waltham, MA, USA).

#### 2.11.2. Apoptosis Assay

The apoptosis was evaluated in vitro by immunofluorescence (IF) with DAPI/Annexin-V or FCM Annexin-V/PI assay (BD Pharmingen, San Diego, CA, USA). AsPC−1, MIAPaCa-2 and PANC−1 cell lines were treated as described for the cell cycle analysis. Afterwards, the medium was removed, and cells were collected and centrifuged at 1500× *g* rpm for 5 min, washed twice with cold PBS without Ca^2+^ and Mg^2+^, resuspended in binding buffer, and stained with Annexin V-FITC/PI for AsPC−1, according to the instructions. MIAPaCa-2 and PANC−1 cells were stained with Annexin V-FITC diluted in binding buffer, washed twice in PBS 1×, and the pellet was resuspended in Vectashield with Dapi (Vector Laboratories, Burlingame, CA), dropped on slides, and covered with coverslip for microscope examination. Apoptotic cells were evaluated by FCM analysis (AsPC−1 cell line) with an Attune NxT Acoustic Focusing Cytometer (Thermo Fisher Scientific, Waltham, MA, USA) or by IF on fluorescence microscope (Leica, Wetzlar, Germany) (MIAPaCa-2 and PANC−1 cell lines).

#### 2.11.3. γ. H_2_AX Analysis

For the analysis of the DNA damage induced by the tested compounds, cells were treated and processed as described for the cell cycle analysis until the fixation step. Fixed cells were then processed as previously described [44,45]. The isotype control (ctrl), purified mouse IgG1 Isotype Control was utilized, the primary antibody was phospho-Histone H2AX (Ser−139) antibody (Millipore, Billerica, MA, USA), and the secondary was the goat anti-mouse IgG (H&L) fluorescein-conjugated, affinity-purified secondary antibody (BD Pharmingen, San Diego, CA, USA). The FCM analysis was performed by Attune NxT Acoustic Focusing Cytometer (Thermo Fisher Scientific, Waltham, MA, USA) and analyzed by using the Attune NxT Analysis Software (Thermo Fisher Scientific, Waltham, MA, USA).

### 2.12. Statistical Analysis

Results were expressed as mean ± SD of three independent experiments and analyzed using GraphPad Prism vers 5.0 (GraphPad, San Diego, CA, USA) to calculate the significance between groups using paired Student’s *t*-test method (two-tailed). For the analysis of variance, statistical significance was calculated by using a two-way ANOVA followed by the Bonferroni post hoc tests (GraphPad Prism vers 5.0). Statistically significant differences are set at probabilities of * *p* < 0.05, ** *p* < 0.01, and *** *p* < 0.001.

## 3. Results and Discussion

### 3.1. Synthesis and Characterization of Copolymers

The development of the double targeted DDSs, TpHResMic, preliminarily envisaged the synthesis of three different block copolymers: PLGA-PEG, PLGA-PLL-DMA, and PLGA-PEG-AE105.

**Figure 2:**
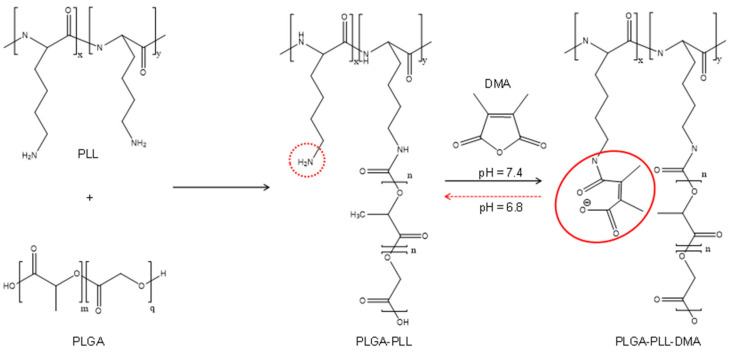
Scheme of synthesis of the PLGA-PLL-DMA copolymer. The continuous red line ring evidences the formation of β-carboxyamide bond which can be hydrolyzed at pH 6.8 in NH_2_ (red dotted line ring) and DMA.

In detail, PLGA was chosen because of its high biocompatibility and biodegradability in aqueous media, which occurs via hydrolytic degradation of the ester linkages present in the polymer chain [46]. PEG is extremely biocompatible and no accumulation in tissue occurs especially for low molecular weight chains. Thanks to its hydrophilicity, PEG polymers and copolymers can be used to stabilize nanoparticles in aqueous media, increase solubility, and avoid aggregation by steric hindrance in production, storage, and application [47]. Moreover, this polymer could confer stealth properties to micelles, and this is a great advantage to avoid the absorption by reticulo endothelial system (RES). PLL is an amino acid, and its NH_2_ groups are positively charged both at pH 6.8 and a pH 7.4. These groups are able to bind by electrostatic absorption to the negatively charged cells surface [48]. DMA was chosen to bind quantitatively to NH_2_ groups of PLL forming β-carboxyamide groups and thus masking their positive charge. This masking, whose consequence is the negative surface charge, could allow to micelles to prolong their permanence in the bloodstream [49] but would reduce the specific cell entry due to the electrostatic interaction between the cell membrane and the micelles. In fact, at normal physiological pH 7.4, amide bond is stable and only when the system is in pH 6.8 conditions (as in PDAC TME) the β-carboxyamide bond could be quickly hydrolyzed, allowing the exposure of NH_2_ groups to positively charge PLL, and consequently, cause cell internalization through the endocytosis pathway [50]. In Figure 2, proper relief has been given to the conjugation step of PLGA with PLL and DMA and specifically to the β-carboxyamide bond between PLL and DMA (continuous red line ring), as it is susceptible to hydrolysis in -NH_2_ (red dotted line ring) and DMA at pH 6.8, thus conferring pH responsiveness to the copolymer PLGA-PLL-DMA.

The syntheses of the copolymers were conducted following the protocols described in the the Supplementary S2. Materials and Methods [51,52,53,54], and to confirm the successful conjugation reactions, the obtained PLGA-PEG and PLGA-PLL-DMA copolymers were characterized by ^1^H-NMR and FT-IR analysis, while the conjugation of PLGA with PEG-AE105 was verified by UV spectroscopy (see the Supplementary S3. Results and discussion, Figure S3).

### 3.2. Determination of Critical Micelle Concentration

**Figure 3:**
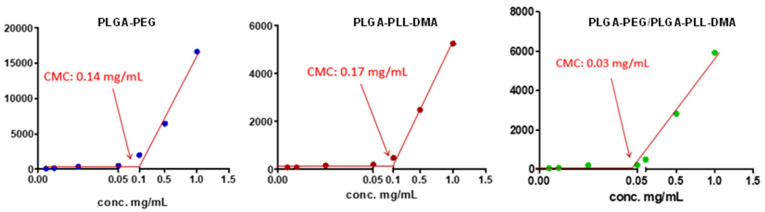
Scattered light intensity graph (in kilo counts per second—Kcps) obtained for the various concentrations of PLGA-PEG, PLGA-PLL-DMA, and the mixture 1:1 (*w*/*w*) of PLGA-PEG/PLGA-PLL-DMA prepared in deionized water. The intersection of the two lines corresponds to the critical micellar concentration.

In order to verify the effective ability of the copolymer mixture to organize itself in such a way as to produce micelles, the CMC was determined both for the single copolymers PLGA-PEG and PLGA-PLL-DMA and for the mixture of them useful for forming the pH-responsive micelles. The dynamic light scattering (DLS) technique was used as analysis methodology for the determination of the CMC [40,41]. Plotting the values of the measured intensities (Kcps) as a function of the polymer concentration, it can be seen that these gradually decrease as the dilution of the micellar suspensions increases (Figure 3). From the interpolation of the lines passing through the points, and from the determination of the intersection point of these lines, it was possible to determine the value of CMC in water for copolymers both in single and in 1:1 (*w*/*w*) mixture. In particular, regarding the PLGA-PEG copolymer, the calculated CMC was 0.14 mg/mL, and, for the PLGA-PLL-DMA copolymer, the calculated CMC value was 0.17 mg/mL. However, the CMC value determined for the PLGA-PEG/PLGA-PLL-DMA mixture was 0.03 mg/mL, almost an order of magnitude lower than those of the single copolymers, which is a sign that the mixture of the two copolymers caused the system to organize itself to form micelles at lower concentrations.

### 3.3. Preparation and Characterization of Micelles

The co-flow geometry was adopted for NoRes, pHResMic, and TpHResMic micelles generation by microfluidics. Using this geometry, the inner fluid (organic phase) passed through the internal capillary, while the outer fluid (aqueous phase) moved along the same direction between the internal and external capillaries. The results of the obtained micelles in terms of particle size, PDI, ζ-potential, and EE% values are summarized in Table 2. All formulations had dimensions below 170 nm with narrow distribution range, which was highlighted by the low poly-dispersion index value (PdI < 0.2). It has been widely demonstrated in the literature that the production of nanoparticles using microfluidics can produce smaller particles than those produced by conventional methods; indeed, the mixing in the microfluidic device is faster than the time needed to verify nucleation and growth of the nanoparticles and results in the formation of more and smaller particles [25,55]. Very small nanoparticles with an average diameter of about 60-70 nm are quickly excreted in vivo, while larger nanoparticles (200 nm or more) can be seized by the liver or spleen; therefore, only nanoparticles with a diameter between 70 and 200 nm, as polymeric micelles, are optimal for in vivo applications [56,57]. Moreover, since the pores of tumor vascular walls have typical junction sizes, whose range is about 200−1200 nm, compared with the tight endothelial junctions of normal vessels (5−10 nm), it is conceivable that appropriately sized micelles are allowed to extravasate into solid tumors and to accumulate in tumor tissue, thanks to the lack of functional lymphatic drainage. Thus, particle size <200 nm is a benefit for micelles because it reduces liver absorption, extends circulation time in the blood, and enhances bioavailability [58]. More recently, nanoparticle systems capable of converting their surface charge in function of pH medium variations have been promoted. Their success is associated with an improved ability to adhere to tumor cells and trigger drug release [49]. As can be seen in Table 2, the NoResMic at pH 6.8 had a ζ-potential of 16.60, which at pH 7.4 became 20.60, probably due to protonation of the amine groups. On the contrary, for pHResMic and targeted TpHResMic, the ζ-potential values changed from −19.30 and −15.64 to 25.50 and 13.70, respectively. These values highlighted that, at pH 7.4, the amino groups were stable, and only when the system was at pH 6.8 did the β-carboxamide bond suffer rapid hydrolysis, allowing exposure of the positively charged NH_2_ groups of the PLL; this confirms that the system responded to pH variations by changing its surface charge from negative to positive. From Table 2, it is also possible to see an increase in the size of the TpHResMic, and this growth was probably related to the use of a third block copolymer (PLGA-PEG-AE105) for this preparation, which, compared with the other two systems, rightly led to an increase in size.

To explore the antitumor efficacy of micelles, Gem was chosen as the model drug and loaded into TpHResMic (Gem@TpHResMic) and pHResMic (Gem@pHResMic). The EE % was calculated as the amount (*w*/*w* %) of the drug incorporated in the core of micelles with respect to the starting drug amount employed for the preparation of the nano-vector. A good value of EE% was achieved for Gem@TpHResMic (29.00% ± 1.22) and Gem@pHResMic (22.00% ± 2.10), allowing the performance of the in vitro biological assays.

**Figure 4:**
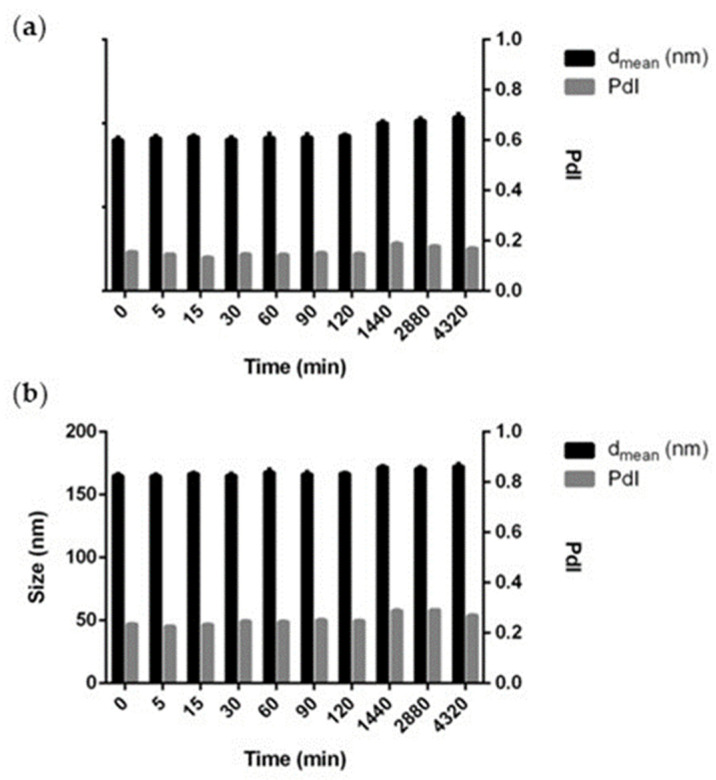
Stability studies of pHResMic (**a**) and TpHResMic (**b**): hydrodynamic diameter and PdI index in PBS pH 7.4 at 37 °C after 72 h. Mean ± SD are reported, *n* = 3.

The stability studies were conducted on pHResMic and TpHResMic in PBS at pH 7.4 by monitoring their particle size using DLS. The micelles possessed good colloidal stability with no evidence of aggregation. In fact, as illustrated in Figure 4, both preparations were stable up to 24 h, with a slight increase in size at 72 h.

**Figure 5:**
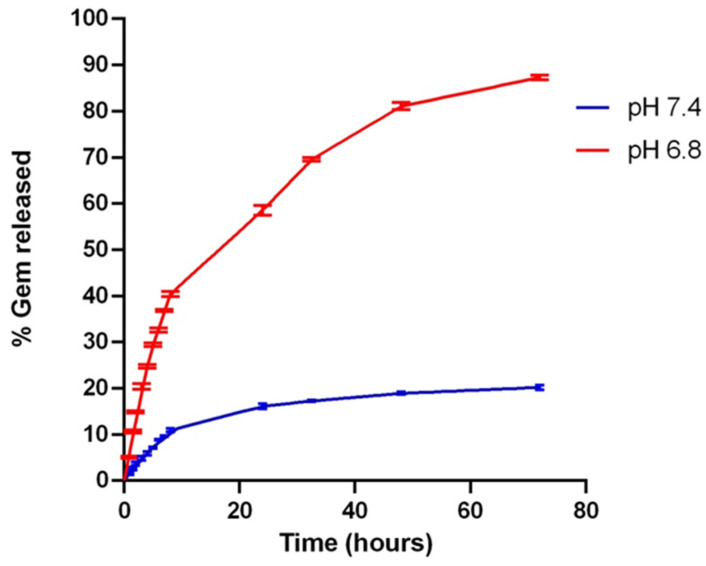
In vitro release profiles of Gem from TpHResMic in PBS at 37 °C at two different pH (6.8 and 7.4). Results are reported as mean ± SD, *n* = 3.

An in vitro drug release study was performed on Gem@TpHResMic, using Franz-diffusion cells at 37 °C in PBS (pH 6.8 and 7.4). As shown in Figure 5, the Gem release from Gem@TpHResMic at pH 7.4 was relatively slow, in fact, only 10.56% Gem was released within the initial 8 h, and it reached a plateau with a release of 15.67% for 24 h. However, Gem release was significantly supported when the pH was decreased. The release profiles at pH 6.8 in Figure 5 revealed a burst release of Gem (40.22%) in 8 h. After incubation for 72 h, the Gem release rate at pH 6.8 were even up to 87.88% in contrast to 20.78% released at pH 7.4. These results showed that the release of the designed micellar system depended on the pH values of the media, namely the release in an acidic environment was higher than in a neutral one. This behavior could be related to the hydrolysis of the amide bonds at pH 6.8 and thus to the exposure of the positive charge, which increased the electrostatic exposure between the cationic nanoparticles and the positively charged drugs, thus triggering its release.

### 3.4. In Vitro Studies

#### 3.4.1. uPAR Expression Evaluation on Plasma Membrane of Pancreatic Cancer Cell Lines

The expression of uPAR on PDAC cell lines was analyzed in order to identify the most suitable cellular models for the evaluation of the efficacy of the Gem-active delivery system, which directly binds this receptor.

**Figure 6:**
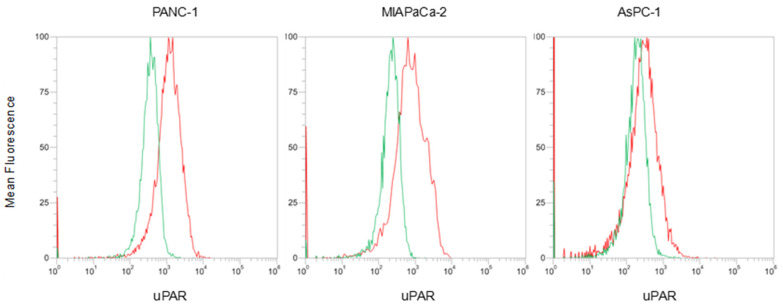
FCM histograms showing the evaluation of uPAR expression on membrane of PDAC cells. Green—cellular autofluorescence; red—uPAR-positive cells.

FCM analysis showed that uPAR is more expressed in PANC−1 and MIAPaCa-2 than in AsPC−1, as reported in Figure 6, in which the right shift of the histograms is representative of a higher uPAR expression.

#### 3.4.2. Evaluation of pH-Mediated Internalization of pH-Responsive Micelles

In all 2D models of PDAC, and depending on the pH environment transition from pH 7.4 to 6.8, the ability of the pH-responsive micellar system to induce the chemical changes necessary to increase tumor cells’ endocytotic internalization was demonstrated by uptake studies. They were carried out incubating PDAC cells with BDP@pH-responsive micelles at two different temperatures (37 °C and 4 °C) and in cell culture media different for pH conditions (7.4 and 6.8), only for the duration of the experiment (1 h), to avoid the insurgence of false positive results due to the specific binding of BDP dye to the documented increased lipid droplets in cells grown in acidic conditions [58]. FCM analysis, conducted after incubation at 37 °C, allowed the quantification of BDP fluorescence relative to both membrane-bound and intracellular micelles, while, after 4 °C incubation, all the endocytotic mechanisms were blocked, and only the cell membranes’ associated fluorescence was detectable [43,59].

**Figure 7:**
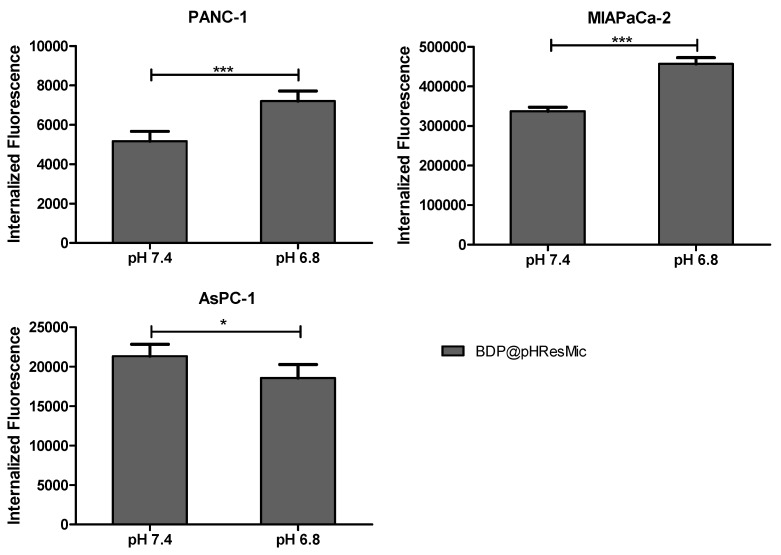
Histogram plots showing the results of uptake studies of pHResMic on 2D model of PDAC cells. The histograms report the amounts of internalized BDP@pHResMic, expressed as the difference between mean fluorescence at 37 °C and at 4 °C registered values, obtained after 1 h incubation of cells with micelles at pH 7.4 or pH 6.8. Histogram bars represent the mean ± SD of three independent experiments. (* *p* < 0.05, *** *p* < 0.001).

The histograms in Figure 7 show the amounts of internalized fluorescence calculated by subtracting 4 °C values from 37 °C values for each analyzed cell line. In PANC−1 and MIAPaCa-2 cell lines, a significant increase in pH-responsive micelles internalization at pH 6.8 was observed; it was 1.4-fold higher than at pH 7.4 for both. In AsPC-1 cells, the trend was opposite with an internalization value 0.87, which was lower at pH 6.8, then at pH 7.4.

We hypothesized that AsPC−1 cells failed to activate the pH-responsive micelles as they are less prone to acidify the culture medium than PANC−1 and MIAPaCa-2 cells, according to Wang, who demonstrated that AsPC−1 released lower levels of lactate than the others [60]. To corroborate our idea, we measured the pH of the conditioned media after 72 h of culture of the 3 PDAC cell lines and the pH changed from pH 7.96 to 7.39 in PANC−1, from pH 7.34 to 6.55 in MIAPaCa-2, it did not change in AsPC−1 cells (pH 72 h = 7.96 as T0). Therefore, PANC−1 and MIAPaCa-2 are intrinsically able to acidify their culture media, unlike the AsPC-1 cell line.

#### 3.4.3. Evaluation of uPAR-Mediated Internalization of Targeted pH-Responsive micelles

The ability of the double targeted micelles (TpHResMic), namely the pH-responsive micelles targeted to the uPAR receptor, to exploit the receptor-mediated endocytosis was assessed in 2D and 3D models of PDAC by FCM and fluorescence imaging.

**Figure 8:**
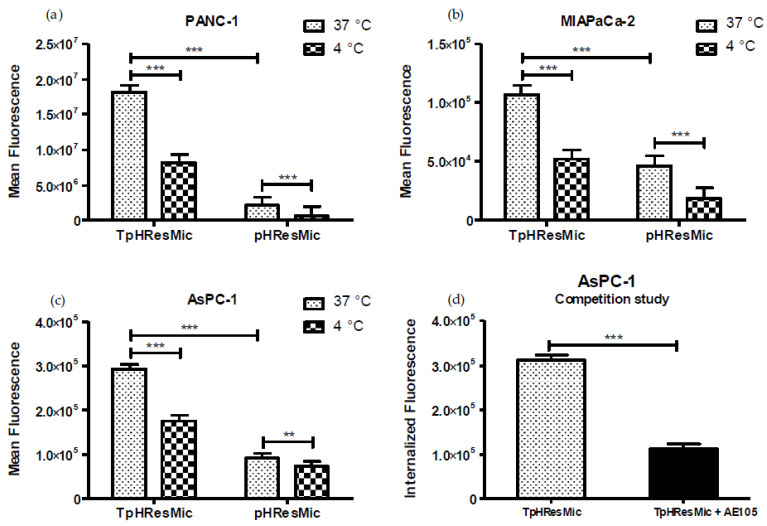
(**a**–**c**) Uptake studies of the targeted (TpHResMic) and no targeted micelles (pHResMic) on 2D model of PDAC cells. The histogram plots report the mean fluorescence values obtained after incubation for 1 h of PDAC cells with BDP@TpHResMic and BDP@pHResMic at pH 6.8 medium and at 37 °C or 4 °C, and refer to three independent experiments. (**d**) The histogram plots report the mean ± SD of the fluorescence values obtained in the competition study of the targeted BDP@TpHResMic, with AE105 peptide in AsPC−1 cell line. (** *p* < 0.01, *** *p* < 0.001).

As evidenced by the higher mean fluorescence values reported in the histograms in Figure 8, TpHResMic, at pH 6.8 and at 37 °C, were significantly more bound to the cell membrane and internalized than no targeted micelles (pHResMic) in all the three PDAC cell lines, with 8.4-, 2.3-, and 3.2-fold changes for PANC−1, MIAPACa-2, and AsPC−1 cell lines, respectively. These results allowed us to confirm the ability of the TpHResMic to recognize the uPAR receptor, thus triggering the receptor-mediated endocytosis pathway, in addition to the endocytosis pathway, due only to the electrostatic interaction of positively charged micelles with the plasmatic membrane. Moreover, since at 4 °C all the endocytotic mechanism are blocked [43,59], the mean fluorescence values recorded after incubation of cells at 4 °C demonstrated a significant reduction compared with 37 °C, both for TpHResMic and pHResMic (Figure 8). At this temperature, it is very plausible that the fluorescence recorded by FCM is due to the micelles bound on the surface of cell membranes; therefore, it is not surprising that, at 4 °C, the amounts of mean fluorescence recorded in all cell lines were higher for cells incubated with the targeted system TpHResMic, compared with those incubated with the pH-responsive and non-targeted system (pHResMic). The differences between all values at 37 °C minus those at 4 °C, provide the internalized fluorescence amount (1.1 × 10^5^, 5.5 × 10^4^, and 9.9 × 10^6^ for AsPC−1, MIAPaCa-2, and PANC−1, respectively) and the ratio between the internalized fluorescence at 37 °C and 4 °C were 7.0, 2.0, and 7.2 in the same cells, respectively. This analysis pointed out that even if AsPC−1 and PANC−1 cell lines have a different ability to acidificate the microenvironment, the double active drug targeting strategy of TpHResMic allows a similar uptake of the micelles into both cell lines, while MIAPaCa-2 cells showed a reduced efficacy of internalization.

To confirm that the increase in the uptake in the AsPC−1 cells was actually linked to receptor recognition and not to a no-specific binding of the loaded BDP to the lipid droplets inside the cells [58], we performed also a competition study, the results of which are reported in Figure 8d. It was evident that the competition with the selective uPAR ligand AE105 strongly reduced the amount of the cells’ internalized fluorescence with a decrease of 2.8-fold, referred to the experiment conducted with no competition.

**Figure 9:**
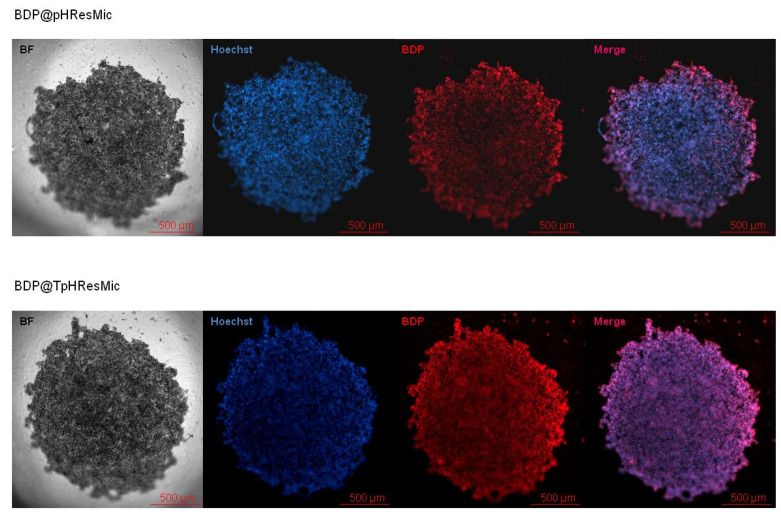
Fluorescence uptake studies on 3D model of PANC−1 cells. The upper panel shows the bright field (BF) image of the spheroid, the fluorescence intensities of Hoechst (blue—nuclei), BDP (red—BDP@pHResMic), and a merge of them; the lower panel shows the bright field (BF) image of the spheroid, the fluorescence intensities of Hoechst (blue—nuclei), BDP (red—BDP@TpHResMic), and a merge of them. Scale bar 500 μm.

In order to evaluate the ability of TpHResMic vs. pHResMic to penetrate a more complex tumor model, we generated 3D model of PDAC with PANC−1 cell line. The PANC−1 spheroids, after 5 days growth, were incubated with TpHResMic and pHResMic for 2 h. The binding and the internalization of micelles was evaluated by fluorescence imaging and the representative images are reported in Figure 9. It was clear an intraspheroids localization of micelles (red) for both the preparations, with a higher fluorescence intensity in the core for the uPAR-targeted system (TpHResMic) than that for the not targeted system (pHResMic) (mean fluorescence intensity of BDP on the central section of the spheroids: 1475.671 vs. 302.530, for TpHResMic and pHResMic, respectively).

#### 3.4.4. Evaluation of the Antiproliferative Effect of TpHResMic and pHResMic in 2D Models of Pancreatic Cancer

**Figure 10:**
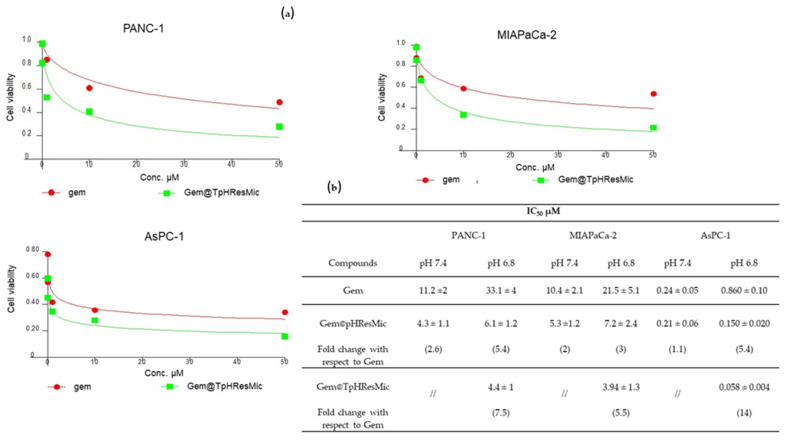
(**a**) Dose/effect plots of the mean of three different cell viability experiments, conducted in PDAC cell lines incubated for 24 h + 48 h w.o. with free Gem or Gem@TpHResMic. (**b**) IC_50_ values of PDAC cell lines treated with all treatment conditions; the data are reported as the mean of three independent experiments.

The cell proliferation study was performed in the 2D PDAC models at pH 7.4 and 6.8 to assess the antiproliferative effect of Gem when loaded in both Gem@TpHResMic and Gem@pHResMic, and in comparison with free Gem. The empty targeted micelles were also tested to understand the intrinsic cytotoxicity of the carrier, which showed no cytotoxic effect in the range of concentrations investigated (data not shown). In Figure 10b, the IC_50_ values obtained performing the MTT assay after the incubation of cells for 24 h are reported, with each compound followed by 48 h washing out (w.o.) in fresh medium. As representative of the proliferation experiments, the dose/effect plots of PDAC cells after treatment with Gem@TpHResMic and the free Gem, in pH 6.8 media, are reported in Figure 10.

Gem@pHResMic showed greater efficacy than Gem at pH 6.8 compared with pH 7.4 in all PDAC models. Indeed, the IC_50_ values were reduced of about 5-, 2-, and 1.5-fold in AsPC−1, PANC−1, and MIAPaCa-2, respectively. At pH 6.8, much greater efficacy was shown by Gem@TpHResMic, with respect to the free drug, with an increase of 14-, 7.5-, and 5.5-fold in AsPC−1, PANC−1, and MIAPaCa-2, respectively. Therefore, all the PDAC models showed higher sensitivity to Gem@TpHResMic, in agreement with the micelles uptake characterization. In PANC−1 and MIAPaCa-2 cell lines, Gem@TpHResMic mainly employed the acidic environment to ensure a slow intracellular release of Gem, leading to a longer lasting antiproliferative effect, compared with the free Gem; while in AsPC−1 cells, they took advantage of the selective binding to uPAR to get closer to the cells and slowly release the drug after their internalization.

**Figure 11:**
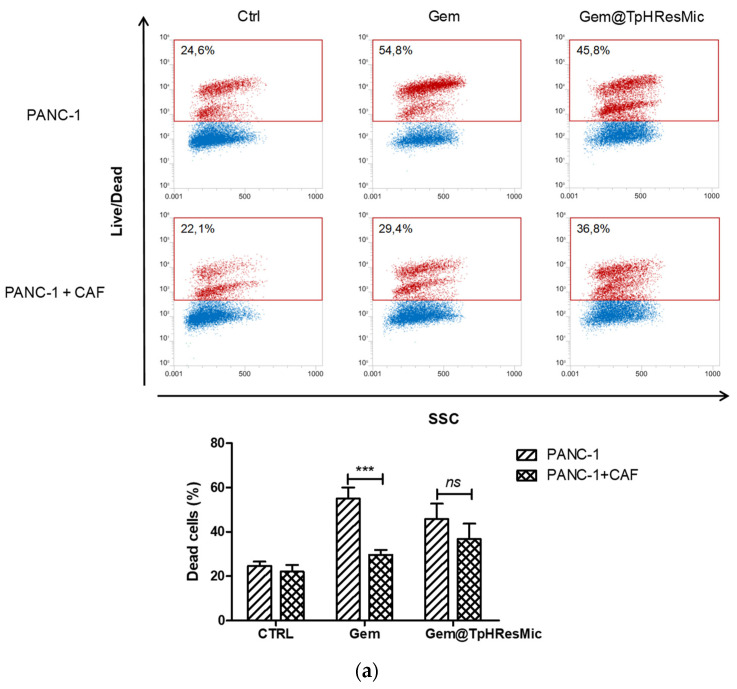

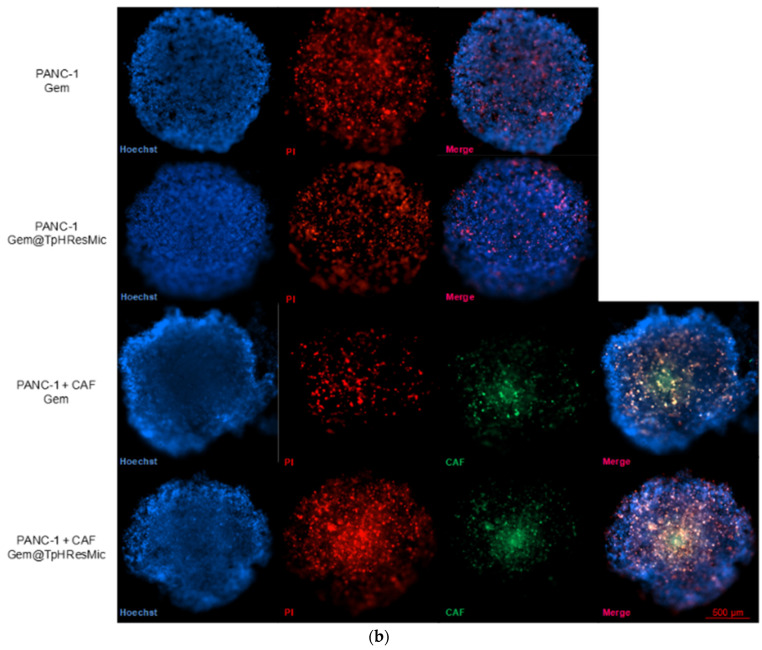
(**a**) Representative FCM dot plots of viability analysis conduct on PANC−1 and PANC−1/CAF spheroids after treatment with Gem@TpHResMic or free Gem. The histograms plots show the % of dead cells in all conditions, reported as the mean ± SD of three independent experiments. (**b**) Representative fluorescence images of viability evaluation in PANC−1 or PANC−1/CAF spheroids (blue—nuclei; green—CAF; red—dead cells). *** *p* < 0.001. Scale bar 500 μm.

Furthermore, the antitumor activity of free or Gem@TpHResMic was studied both in homospheroids of the PANC−1 cell line and in co-cultured spheroids with CAF to simulate the TME. For this purpose, both types of spheroids were treated with the Gem@TpHResMic or with the free Gem for 24 h, followed by 48h drug w.o., and at the end of the treatment the evaluation of the drug’s cytotoxicity was determined by FCM. We found that the percentage of dead cells in the spheroids of tumor cells was quite similar after the treatment with Gem@TpHResMic or with the free Gem (45.8 ± 7,1 vs. 54.8 ± 5.0). The presence of CAF in spheroids caused a strong and significant reduction in the efficacy of the free Gem, while it reduced to a lesser extent the effectiveness of Gem-loaded micelles (Figure 11a). The images in Figure 11b further show that the micellar system allowed for more effective targeting of TME and led to greater retention of the efficacy of Gem, as demonstrated by the increase in PI-stained cells in the CAF/tumor cells spheroids (Figure 11b).

#### 3.4.5. Evaluation of the Antitumor Activity of Free Gem and Gem@TPHResMic through the Analysis of γH2AX, the Cell Cycle Perturbation and the Apoptosis Induction

In order to investigate how the intracellular activity of Gem changes when administered in this new formulation, we evaluated the activation of the DNA damage response (DDR) [45], by analyzing the phosphorylation of the Ser−139 residue of the histone variant H2AX (γH2AX), which marks the sites of DNA damage, and the cell cycle modulation and the apoptosis induction by free Gem and Gem-loaded micelles (Gem@TPHResMic). The experiments for evaluating the extent of DNA damage (γH2AX) by both treatments were performed after 24 h of drugs exposure.

**Figure 12:**
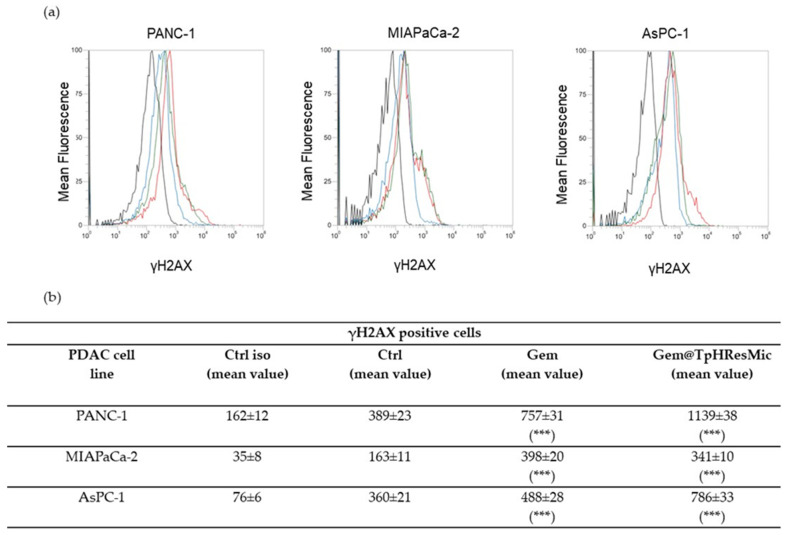
(**a**) Representative FCM histograms showing the γH2AX positive PDAC cells after treatment for 24 h at pH 6.8 with free Gem or Gem@TpHResMic (black line—isotype ctrl; blue line—ctrl; green line—Gem; red line—Gem@TpHResMic). (**b**) Mean values of γH2AX-positive PDAC cells in all treatment conditions are reported as the mean ± SD of the three independent experiments (*** *p* < 0.001 as respect to untreated cells; Ctrl—untreated cells).

The results of the analysis showed, in AsPC−1 cell lines, a significant increase in γH2AX of about 2- and 4-fold, when free Gem and Gem@TpHResMic were added to cells, respectively (Figure 12). Likewise, in PANC−1 cell line, we observed an almost 3-fold increase in γH2AX when treated with Gem@TpHResMic and a 2-fold increase after free Gem treatment, compared with untreated cells. Unlike the previous cell lines, in MIAPaCa-2 cells, both the free Gem and Gem@TpHResMic caused a similar phosphorylation of H2AX, of about 2-fold, with respect to untreated cells. These data suggested that Gem@TpHResMic had a greater potential to damage DNA than free Gem.

**Figure 13:**
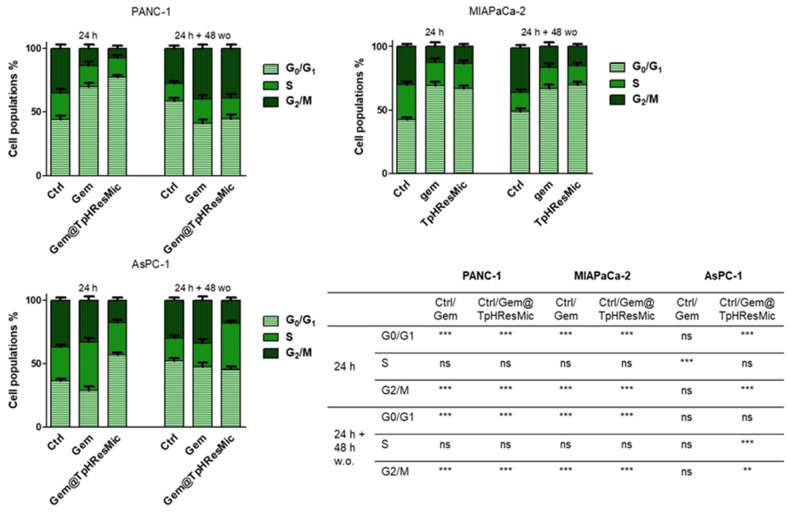
Cell cycle perturbation of PDAC cells by free Gem and Gem@TpHResMic at pH 6.8 after 24 h and 24 h + 48 h w.o. The modulation of cell cycle phases was evaluated by FCM analysis after staining the cells with propidium iodide. The bar graphs show % cell population distributions in G0/G1, S, or G2/M phases, reported as the mean ± SD of the three independent experiments, and the statistical significances are reported in the table (** *p* < 0.01, *** *p* < 0.001).

To evaluate whether the massive Gem@TpHResMic-dependent accumulation of DNA damage resulted in mitotically arrested cells, we evaluated the cell cycle perturbation after 24 h of treatments and after 24 h followed by 48 h drug w.o. In agreement with this hypothesis, we found that 24 h Gem treatment induced S phase cell cycle arrest in AsPC−1 cell line, according to checkpoint activation following DNA damage. However, after 48 h of drug w.o., the AsPC−1 cells recovered a replication rate similar to untreated cells, probably because they escaped mitotic arrest as a consequence of a checkpoint adaptation, which allowed cells to restore cell division [61] (Figure 13). The analysis of cell cycle of AsPC−1 cells, treated with Gem@TpHResMic, showed instead a significant arrest at G0/G1 stage after 24 h of treatment, and a significant arrest at S phase following 24 h and 48 h of drug w.o. Additionally, as the proportion of cells in S phase increased, this accumulation was associated with a reduction in G2/M, lending credence to the notion that Gem@TpHResMic generated heavily damaged cells, which during the next cell division failed to escape mitotic arrest and they died. In the MIAPaCa-2 cell line, the Gem@TpHResMic exerted an effect similar to the free Gem, by causing a prolonged accumulation of cells in the G0/G1 phase along 48 h of drug wash out and a consequent reduction in S and G2/M phase, which is consistent with cells arrested in interphase, because they have failed mitosis and presumably will become senescent [61]. Similar to the previous cell lines, in PANC−1 cells, both the free Gem and Gem@TpHResMic arrested the cells in the G0/G1 phase after 24 h and accumulated them in the G2/M phase after 24 and 48 h of w.o. drug, consistent with cells that can overcome S phase arrest but become aneuploid and die before commencing subsequent cell division, because of massive DNA damage (γH2AX increase)—especially induced by Gem@TpHResMic. Collectively, these data suggested that Gem@TpHResMic had a greater ability than free Gem to generate mitotically arrested cells that eventually entered cellular senescence (arrested in G0/G1) or die (arrested in G2/M).

**Figure 14:**
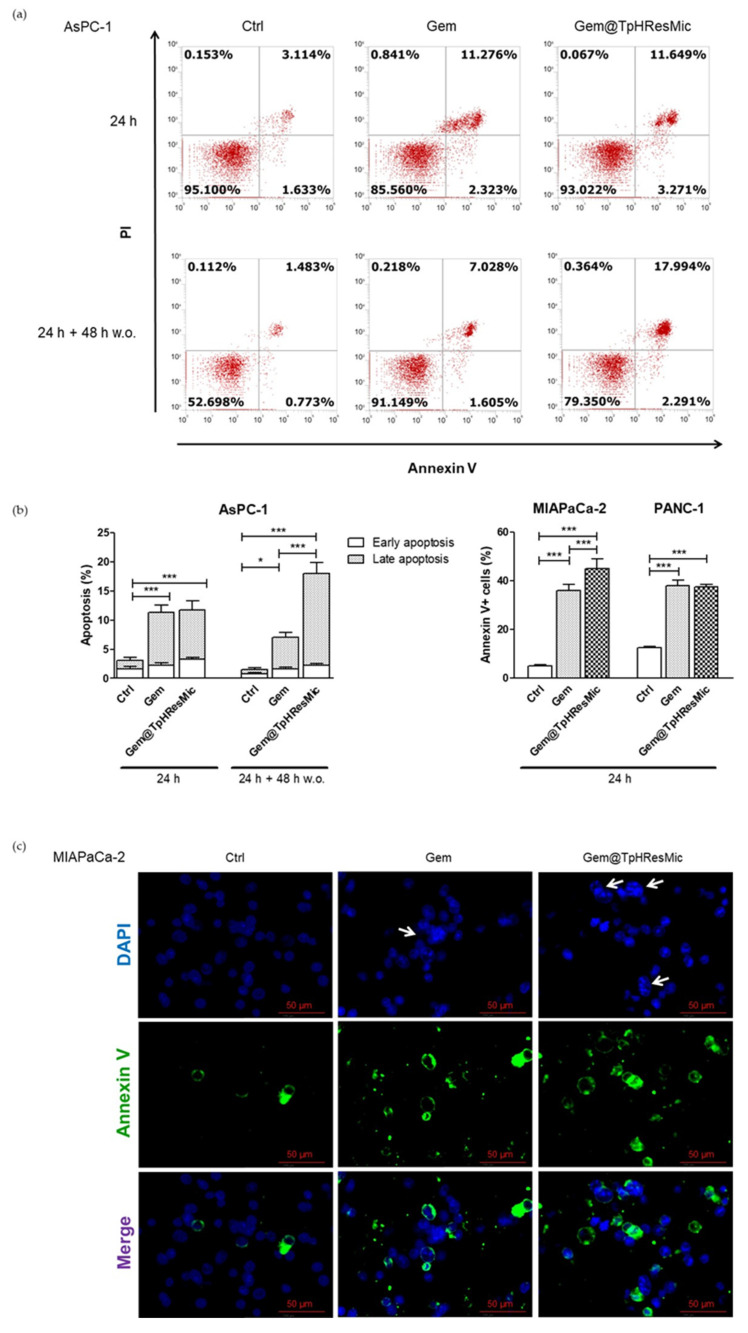
Evaluation of induction of apoptosis in PDAC cell lines treated with Gem and Gem@TpHResMic. (**a**) Representative dot plots showing FCM analysis in AsPC−1 cell line. (**b**) Bar graphs summarizing the results of the assays, as the mean ± SD of the three independent experiments (* *p* < 0.05, *** *p* < 0.001). (**c**) Representative IF images of the MIAPaCa-2 cell line, showing nuclei features (white arrows show polynucleated cells) and Annexin V-positive cells (blue—DAPI-stained nuclei; green—Annexin V).

To gain insights on the apoptotic potential of Gem@TpHResMic in mitotically arrested cells, we evaluated the apoptosis induction in each cell line after treatment with free Gem and Gem-loaded micelles. Because, in the AsPC−1 cell line, we observed a differential effect in terms of cell cycle modulation by free GEM and Gem@TpHResMic after 48 h drug w.o., we analyzed the induction of apoptosis at both 24 h and at 24 h and 48 h of drug w.o through AnnexinV/PI staining and FCM analysis. The data showed that 24 h treatment, both with free Gem and Gem@TpHResMic, had similar apoptotic potential, resulting in an increase of about 14% and 15% AnnexinV/PI positive cells, respectively, compared with untreated cells; instead, according to cell cycle modulation, the results of the analysis performed after 24 h and 48 h of w.o. evidenced that the pro-apoptotic effect of free Gem was reduced (about 9% vs. untreated cells), and that of Gem@TpHResMic was increased (by about 20%, vs. untreated cells). Both FCM dot plots and data analysis are reported in Figure 14a,b. As we hypothesized that, in both PANC−1 and MIAPaCa-2 cell lines, Gem@TpHResMic would generate mitotically arrested cells, we performed the immunofluorescence examination of the DAPI-stained nuclei. Additionally, because prolonged mitosis may result in mitotic slippage and escape from death, we also stained the cells with AnnexinV-FITC antibody to reveal induction of apoptosis. The images in Figure 14c show that Gem@TpHResMic induced a higher accumulation of polynucleated cells, typical of cells arrested in interphase, because they failed cellular division, along with Annexin V-positive cells. This demonstrated that the cells did not survive the treatment with the Gem-loaded micelles; additionally, as shown by the Annexin V-positive cells quantification (Figure 14b), the extent of apoptosis was stronger than that induced by free Gem in the MIAPaCa-2 cell line, and was similar to free Gem in the PANC−1 cell line (Figure S1).

## 4. Conclusions

One of the causes of the failure of classical chemotherapeutic regimens for PDAC is the drug resistance of the cancer, which is due to the intrinsic characteristics of the tumor and its microenvironment. To overcome this aspect, and thanks to an innovative microfluidic technological approach, we formulated the model anticancer drug Gem as a polymeric micellar delivery system, responsive to pH variability in the tumor microenvironment. AE105 peptide was also introduced on the surface of these pH-responsive micelles as a targeting moiety to the uPAR receptor, which was overexpressed in PDAC cells. A microfluidics technique allowed us to obtain homogeneously dispersed micelles with a narrow size range and good colloidal stability, with no evidence of aggregation, as confirmed by the stability study. The peculiar micelles composition allowed controlled Gem release kinetics in response to pH stimuli. The double targeted Gem delivery system was shown to be more internalized in the PANC−1 and MIAPaCa-2 cell lines, thanks to the pH-mediated active targeting strategy. On the other hand, in AsPc−1 cells, the active targeting strategy was uPAR receptor-mediated, which certainly influenced the increase in cell internalization of the micellar system and resulted in greater antitumor efficacy, as confirmed by the competition study with the selective uPAR ligand. From this point of view, the double targeting strategy proved to be successful in the hypothesis of being able to reach, simultaneously and selectively, different types of cancer cells. The results obtained from the 2D and 3D models of the pancreatic cancer cells and in the more physiologically relevant system of PDAC—obtained by co-culturing CAF and pancreatic tumor cells in 3D—confirmed the high level of cell internalization, the ability to control the release of the loaded drug, and a relevant antitumor efficacy, compared with unformulated Gem. In fact, the Gem@TpHResMic had a greater potential to damage DNA than the free Gem, and this was reflected in the increased induction of apoptosis and persistent perturbation of the cell cycle, which may prove to be a useful strategy to target those cancer cells that use mitotic slippage as a drug rescue mechanism. Collectively, the results obtained in this study encourage further investigations, especially through an in vivo study, in order to be able to consider this targeted pH-responsive drug delivery system as a candidate for a new therapeutic approach to improve treatment response, not only in pancreatic cancer, but also in other types of cancer with similar characteristics.

## Figures and Tables

**Table 1:** Description of the polymeric composition of micelles. +: present in the formulation, −: not present in the formulation.

Micelles	PLGA-PEG	PLGA-PLL	PLGA-PLL-DMA	PLGA-PEG-AE105
NoResMic (Non-responsive micelles)	+	+	−	−
pHResMic (pH-responsive micelles)	+	−	+	−
TpHResMic (Targeted pH-responsive micelles)	+	−	+	+

**Table 2:** Intensity average hydrodynamic diameter and corresponding PdI, determined by DLS, ζ-potential value, and drug encapsulation efficiency (EE%) of all prepared micelles.

Micelles Samples	d_mean_		PdI		ζ-Potential		EE%
	pH 7.4	pH 6.8	pH 7.4	pH 6.8	pH 7.4	pH 6.8	
NoResMic	102.70 ± 5.64	92.24 ± 4.54	0.16 ± 0.02	0.14 ± 0.05	16.60 ± 4.53	20.60 ± 3.32	/
pHResMic	90.20 ± 1.26	86.47 ± 1.22	0.18 ± 0.03	0.15 ± 0.05	−19.30 ± 3.33	25.50 ± 1.59	/
TpHResMic	165.60 ± 1.24	130.60 ± 1.08	0.23 ± 0.01	0.26 ± 0.02	−15.64 ± 0.86	13.70 ± 1.87	/
Gem@pHResMic	92.10 ± 1.12	104.30 ± 1.23	0.15 ± 0.02	0.16 ± 0.04	−15.20 ± 2.09	22.80 ± 1.50	22.00% ± 2.10
Gem@TpHResMic	134.30 ± 3.74	124.40 ± 3.47	0.22 ± 0.05	0.19 ± 0.05	−13.80 ± 1.69	16.80 ± 0.70	29.00% ± 1.22

## Data Availability

All data are available at the laboratory of Experimental Pharmacology of IRCCS Istituto Tumori Giovanni Paolo II and at the Department of Pharmacy—Pharmaceutical Sciences, University of Bari.

## Supplementary Materials

The following are available online at https://www.mdpi.com/article/10.3390/cancers14010005/s1, Figure S1: Representative IF images of PANC-1 cell line showing nuclei features (white arrows show polinucleated cells) and Annexin V positive cells (blue: DAPI-stained nuclei, green: Annevin V). Supplementary S2: Materials and Methods, Supplementary Results and discussion. Figure S3: ^1^H-NMR (a) and FT-IR (b) spectra of PLGA-PEG a, PLGA-PLL b and PLGA-PLL-DMA c along with the representation of the chemical structure of PLGA-PLL copolymer. UV-Vis Spectra (c) of PLGA-PEG-AE105 (blue) and PEG-AE105 (red).

## References

[1] AIOM. https://www.aiom.it/wp-content/uploads/2020/10/2020_LG_AIOM_Pancreas.pdf.

[2] Siegel R.L., Miller K.D., Jemal A. (2020). Cancer Statistics, 2020. CA A Cancer J. Clin..

[3] Porcelli L., Iacobazzi R.M., Di Fonte R., Serratì S., Intini A., Solimando A.G., Brunetti O., Calabrese A., Leonetti F., Azzariti A. (2019). CAFs and TGF-β Signaling Activation by Mast Cells Contribute to Resistance to Gemcitabine/Nabpaclitaxel in Pancreatic Cancer. Cancers.

[4] Qin C., Yang G., Yang J., Ren B., Wang H., Chen G., Zhao F., You L., Wang W., Zhao Y. (2020). Metabolism of Pancreatic Cancer: Paving the Way to Better Anticancer Strategies. Mol. Cancer.

[5] Chen X., Zhou W., Liang C., Shi S., Yu X., Chen Q., Sun T., Lu Y., Zhang Y., Guo Q. (2019). Codelivery Nanosystem Targeting the Deep Microenvironment of Pancreatic Cancer. Nano Lett..

[6] Vander Heiden M.G., Cantley L.C., Thompson C.B. (2009). Understanding the Warburg Effect: The Metabolic Requirements of Cell Proliferation. Science.

[7] Chang Q., Jurisica I., Do T., Hedley D. (2011). Hypoxia Predicts Aggressive Growth and Spontaneous Metastasis Formation from Orthotopically Grown Primary Xenografts of Human Pancreatic Cancer. Cancer Res..

[8] Chandana S., Babiker H.M., Mahadevan D. (2018). Therapeutic Trends in Pancreatic Ductal Adenocarcinoma (PDAC). Expert Opin. Investig. Drugs.

[9] Conroy T., Hammel P., Hebbar M., Ben Abdelghani M., Wei A.C., Raoul J.-L., Choné L., Francois E., Artru P., Biagi J.J. (2018). FOLFIRINOX or Gemcitabine as Adjuvant Therapy for Pancreatic Cancer. N. Engl. J. Med..

[10] Shukla S.K., Purohit V., Mehla K., Gunda V., Chaika N.V., Vernucci E., King R.J., Abrego J., Goode G.D., Dasgupta A. (2017). MUC1 and HIF-1alpha Signaling Crosstalk Induces Anabolic Glucose Metabolism to Impart Gemcitabine Resistance to Pancreatic Cancer. Cancer Cell.

[11] Dalin S., Sullivan M.R., Lau A.N., Grauman-Boss B., Mueller H.S., Kreidl E., Fenoglio S., Luengo A., Lees J.A., Heiden M.G.V. (2019). Deoxycytidine Release from Pancreatic Stellate Cells Promotes Gemcitabine Resistance. Cancer Res..

[12] Mashayekhi V., Mocellin O., Fens M.H., Krijger G.C., Brosens L.A., Oliveira S. (2021). Targeting of Promising Transmembrane Proteins for Diagnosis and Treatment of Pancreatic Ductal Adenocarcinoma. Theranostics.

[13] Lai E., Puzzoni M., Ziranu P., Pretta A., Impera V., Mariani S., Liscia N., Soro P., Musio F., Persano M. (2019). New Therapeutic Targets in Pancreatic Cancer. Cancer Treat. Rev..

[14] Han W., Ke J., Guo F., Meng F., Li H., Wang L. (2021). Construction and Antitumor Properties of a Targeted Nano-Drug Carrier System Responsive to the Tumor Microenvironment. Int. J. Pharm..

[15] Kashkooli F.M., Soltani M., Souri M. (2020). Controlled Anti-Cancer drug Release through Advanced Nano-Drug Delivery Systems: Static and Dynamic Targeting Strategies. J. Control. Release.

[16] Chen Y., Du Q., Zou Y., Guo Q., Huang J., Tao L., Shen X., Peng J. (2020). Co-Delivery of Doxorubicin and Epacadostat via Heparin coated pH-Sensitive Liposomes to Suppress the Lung Metastasis of Melanoma. Int. J. Pharm..

[17] Wang Y., Wang X., Zhang J., Wang L., Ou C., Shu Y., Wu Q., Ma G., Gong C. (2019). Gambogic Acid-Encapsulated Polymeric Micelles Improved Therapeutic Effects on Pancreatic Cancer. Chin. Chem. Lett..

[18] Gurka M.K., Pender D., Chuong P., Fouts B.L., Sobelov A., McNally M.W., Mezera M., Woo S.Y., McNally L.R. (2016). Identification of Pancreatic Tumors in Vivo with Ligand-Targeted, pH Responsive Mesoporous Silica Nanoparticles by Multispectral Optoacoustic Tomography. J. Control. Release.

[19] Skovgaard D., Persson M., Kjaer A. (2017). Urokinase Plasminogen Activator Receptor–PET with 68 Ga-NOTA-AE105. PET Clin..

[20] Gao G., Li Y., Lee D.S. (2012). Environmental pH-Sensitive Polymeric Micelles for Cancer Diagnosis and Targeted Therapy. J. Control. Release.

[21] Cheng W., Gu L., Ren W., Liu Y. (2014). Stimuli-Responsive Polymers for Anti-Cancer Drug Delivery. Mater. Sci. Eng. C.

[22] Wu W., Luo L., Wang Y., Wu Q., Dai H.-B., Li J.-S., Durkan C., Wang N., Wang G.-X. (2018). Endogenous pH-Responsive Nanoparticles with Programmable Size Changes for Targeted Tumor Therapy and Imaging Applications. Theranostics.

[23] Yu G., Ning Q., Mo Z., Tang S. (2019). Intelligent Polymeric Micelles for Multidrug Co-Delivery and Cancer Therapy. Artif. Cells Nanomed. Biotechnol..

[24] Martins C., Araújo F., Gomes M.J., Fernandes C., Nunes R., Li W., Santos H.A., Borges F., Sarmento B. (2019). Using Microfluidic Platforms to Develop CNS-Targeted Polymeric Nanoparticles for HIV Therapy. Eur. J. Pharm. Biopharm..

[25] Arduino I., Liu Z., Rahikkala A., Figueiredo P., Correia A., Cutrignelli A., Denora N., Santos H.A. (2021). Preparation of Cetyl Palmitate-Based PEGylated Solid Lipid Nanoparticles by Microfluidic Technique. Acta Biomater..

[26] Arduino I., Liu Z., Iacobazzi R.M., Lopedota A.A., Lopalco A., Cutrignelli A., Laquintana V., Porcelli L., Azzariti A., Franco M. (2021). Microfluidic Preparation and in Vitro Evaluation of iRGD-Functionalized Solid Lipid Nanoparticles for Targeted Delivery of Paclitaxel to Tumor Cells. Int. J. Pharm..

[27] Tiboni M., Benedetti S., Skouras A., Curzi G., Perinelli D.R., Palmieri G.F., Casettari L. (2020). 3D-Printed Microfluidic Chip for the Preparation of Glycyrrhetinic Acid-Loaded Ethanolic Liposomes. Int. J. Pharm..

[28] Hussain M.T., Tiboni M., Perrie Y., Casettari L. (2020). Microfluidic Production of Protein Loaded Chimeric Stealth Liposomes. Int. J. Pharm..

[29] Xu Z., Lu C., Riordon J., Sinton D., Moffitt M.G. (2016). Microfluidic Manufacturing of Polymeric Nanoparticles: Comparing Flow Control of Multiscale Structure in Single-Phase Staggered Herringbone and Two-Phase Reactors. Langmuir.

[30] Chastek T.Q., Iida K., Amis E.J., Fasolka M.J., Beers K.L. (2008). A Microfluidic Platform for Integrated Synthesis and Dynamic Light Scattering Measurement of Block Copolymer Micelles. Lab A Chip.

[31] Capretto L., Mazzitelli S., Colombo G., Piva R., Penolazzi L., Vecchiatini R., Zhang X., Nastruzzi C. (2013). Production of Polymeric Micelles by Microfluidic Technology for Combined Drug Delivery: Application to Osteogenic Differentiation of Human Periodontal Ligament Mesenchymal Stem Cells (hPDLSCs). Int. J. Pharm..

[32] Capretto L., Chengx W., Carugo D., Hill M., Zhang X. (2010). Microfluidic Reactors for Controlled Synthesis of Polymeric Micelles. J. Control. Release.

[33] Lu Y., Chowdhury D., Vladisavljević G.T., Koutroumanis K., Georgiadou S. (2016). Production of Fluconazole-Loaded Polymeric Micelles Using Membrane and Microfluidic Dispersion Devices. Membranes.

[34] Nastruzzi C., Capretto L., Mazzitelli S., Brognara E., Lampronti I., Carugo D., Hill M., Zhang X., Gambari R. (2012). Mithramycin Encapsulated in Polymeric Micelles by Microfluidic Technology as Novel Therapeutic Protocol for Beta-Thalassemia. Int. J. Nanomed..

[35] Capretto L., Carugo D., Cheng W., Hill M., Zhang X. (2011). Continuous-Flow Production of Polymeric Micelles in Microreactors: Experimental and Computational Analysis. J. Colloid Interface Sci..

[36] Xu J., Zhang S., Machado A., Lecommandoux S., Sandre O., Gu F., Colin A. (2017). Controllable Microfluidic Production of Drug-Loaded PLGA Nanoparticles Using Partially Water-Miscible Mixed Solvent Microdroplets as a Precursor. Sci. Rep..

[37] Feng Q., Liu J., Li X., Chen Q., Sun J., Shi X., Ding B., Yu H., Li Y., Jiang X. (2016). One-Step Microfluidic Synthesis of Nanocomplex with Tunable Rigidity and Acid-Switchable Surface Charge for Overcoming Drug Resistance. Small.

[38] Albuquerque L.J.C., Sincari V., Jäger A., Konefał R., Pánek J., Černoch P., Pavlova E., Štěpánek P., Giacomelli F.C., Jäger E. (2019). Microfluidic-Assisted Engineering of Quasi-Monodisperse pH-Responsive Polymersomes toward Advanced Platforms for the Intracellular Delivery of Hydrophilic Therapeutics. Langmuir.

[39] Brown L., McArthur S.L., Wright P.C., Lewis A., Battaglia G. (2010). Polymersome Production on a Microfluidic Platform Using pH Sensitive Block Copolymers. Lab A Chip.

[40] Amphoterics N., Einstein S., Dls C., Nano T.Z. (2006). Surfactant Micelle Characterization Using Dynamic Light Scattering. J. Phys. Chem. B.

[41] Zdziennicka A., Szymczyk K., Krawczyk J., Jańczuk B. (2012). Critical Micelle Concentration of Some Surfactants and Thermodynamic Parameters of Their Micellization. Fluid Phase Equilibria.

[42] Iacobazzi R.M., Cutrignelli A., Stefanachi A., Porcelli L., Lopedota A.A., Di Fonte R., Lopalco A., Serratì S., Laquintana V., Silvestris N. (2020). Hydroxy-Propil-β-Cyclodextrin Inclusion Complexes of Two Biphenylnicotinamide Derivatives: Formulation and Anti-Proliferative Activity Evaluation in Pancreatic Cancer Cell Models. Int. J. Mol. Sci..

[43] Iacobazzi R.M., Porcelli L., Lopedota A.A., Laquintana V., Lopalco A., Cutrignelli A., Altamura E., Di Fonte R., Azzariti A., Franco M. (2017). Targeting Human Liver Cancer Cells with Lactobionic Acid-G(4)-PAMAM-FITC Sorafenib Loaded Dendrimers. Int. J. Pharm..

[44] Azzariti A., Colabufo N.A., Berardi F., Porcelli L., Niso M., Simone G.M., Perrone R., Paradiso A. (2006). Cyclohexylpiperazine Derivative PB28, a σ2 Agonist and σ1 Antagonist Receptor, Inhibits Cell Growth, Modulates P-Glycoprotein, and Synergizes with Anthracyclines in Breast Cancer. Mol. Cancer Ther..

[45] Porcelli L., Iacobazzi R.M., Quatrale A.E., Bergamini C., Denora N., Crupi P., Antonacci D., Mangia A., Simone G., Silvestris N. (2017). Grape Seed Extracts Modify the Outcome of Oxaliplatin in Colon Cancer Cells by Interfering with Cellular Mechanisms of Drug Cytotoxicity. Oncotarget.

[46] Yallapu M.M., Gupta B.K., Jaggi M., Chauhan S.C. (2010). Fabrication of Curcumin Encapsulated PLGA Nanoparticles for Improved Therapeutic Effects in Metastatic Cancer Cells. J. Colloid Interface Sci..

[47] Locatelli E., Franchini M.C. (2012). Biodegradable PLGA-b-PEG Polymeric Nanoparticles: Synthesis, Properties, and Nanomedical Applications as Drug Delivery System. J. Nanoparticle Res..

[48] Liu P., Yu H., Sun Y., Zhu M., Duan Y. (2012). A mPEG-PLGA-b-PLL Copolymer Carrier for Adriamycin and siRNA Delivery. Biomaterials.

[49] Huo Q., Zhu J., Niu Y., Shi H., Gong Y., Li Y., Song H. (2017). pH-Triggered Surface Charge-Switchable Polymer Micelles for the Co-Delivery of Paclitaxel/Disulfiram and Overcoming Multidrug Resistance in Cancer. Int. J. Nanomed..

[50] Chen S., Rong L., Lei Q., Cao P.-X., Qin S.-Y., Zheng D.-W., Jia H.-Z., Zhu J.-Y., Cheng S.-X., Zhuo R.-X. (2016). A Surface Charge-Switchable and Folate Modified System for Co-Delivery of Proapoptosis Peptide and p53 Plasmid in Cancer Therapy. Biomaterials.

[51] Li X.-X., Chen J., Shen J.-M., Zhuang R., Zhang S.-Q., Zhu Z.-Y., Ma J.-B. (2018). pH-Sensitive Nanoparticles as Smart Carriers for Selective Intracellular Drug Delivery to Tumor. Int. J. Pharm..

[52] Jeong J.H., Park T.G. (2002). Poly(L-Lysine)-g-poly(D,L-Lactic-Co-Glycolic Acid) Micelles for Low Cytotoxic Biodegradable Gene Delivery Carriers. J. Control. Release.

[53] Margiotta N., Savino S., Denora N., Marzano C., Laquintana V., Cutrignelli A., Hoeschele J.D., Gandin V., Natile G. (2016). Encapsulation of Lipophilic Kiteplatin Pt(iv) Prodrugs in PLGA-PEG Micelles. Dalton Trans..

[54] Annese C., Abbrescia D.I., Catucci L., D’Accolti L., Denora N., Fanizza I., Fusco C., La Piana G. (2013). Site-Dependent Biological Activity of Valinomycin Analogs Bearing Derivatizable Hydroxyl Sites. J. Pept. Sci..

[55] Tahir N., Madni A., Li W., Correia A., Khan M.M., Rahim M.A., Santos H.A. (2020). Microfluidic Fabrication and Characterization of Sorafenib-Loaded Lipid-Polymer Hybrid Nanoparticles for Controlled Drug Delivery. Int. J. Pharm..

[56] Owens D.E., Peppas N.A. (2006). Opsonization, Biodistribution, and Pharmacokinetics of Polymeric Nanoparticles. Int. J. Pharm..

[57] Moghimi S.M., Porter C., Muir I., Illum L., Davis S. (1991). Non-Phagocytic Uptake of Intravenously Injected Microspheres in Rat spleen: Influence of Particle Size and Hydrophilic Coating. Biochem. Biophys. Res. Commun..

[58] Corbet C., Bastien E., De Jesus J.P.S., Dierge E., Martherus R., Linden C.V., Doix B., Degavre C., Guilbaud C., Petit L. (2020). TGFβ2-Induced Formation of Lipid Droplets Supports Acidosis-Driven EMT and the Metastatic Spreading of Cancer Cells. Nat. Commun..

[59] Denora N., Laquintana V., Lopalco A., Iacobazzi R.M., Lopedota A., Cutrignelli A., Iacobellis G., Annese C., Cascione M., Leporatti S. (2013). In Vitro Targeting and Imaging the Translocator Protein TSPO 18-kDa through G(4)-PAMAM–FITC Labeled Dendrimer. J. Control. Release.

[60] Wang F., Liu H., Hu L., Liu Y., Duan Y., Cui R., Tian W. (2018). The Warburg Effect in Human Pancreatic Cancer Cells Triggers Cachexia in Athymic Mice Carrying the Cancer Cells. BMC Cancer.

[61] Haschka M., Karbon G., Fava L., Villunger A. (2018). Perturbing mitosis for Anti-Cancer Therapy: Is Cell Death the Only Answer?. EMBO Rep..

