# Supplementary material for: Microfluidic-Assisted Preparation of Targeted pH-Responsive Polymeric Micelles Improves Gemcitabine Effectiveness in PDAC: In Vitro Insights
Source: Cancers (Basel). 2021 Dec 21;14(1):5. doi: 10.3390/cancers14010005 (PMC8750671; doi:10.3390/cancers14010005)
Supplement: Supplementary file 1 [file cancers-14-00005-s001.zip › cancers-1426251-supplementary.pdf]

cancers

MDPI

Supplementary Materials

Microfluidic-Assisted Preparation of Targeted pH-responsive Polymeric Micelles Improves Gemcitabine Effectiveness in PDAC: In Vitro Insights

Rosa Maria Iacobazzi, Ilaria Arduino, Roberta Di Fonte, Angela Assunta Lopedota, Simona Serratì, Giuseppe Racaniello, Viviana Bruno, Valentino Laquintana, Byung Chul Lee, Nicola Silvestris, Francesco Leonetti, Nunzio Denora, Letizia Porcelli and Amalia Azzariti

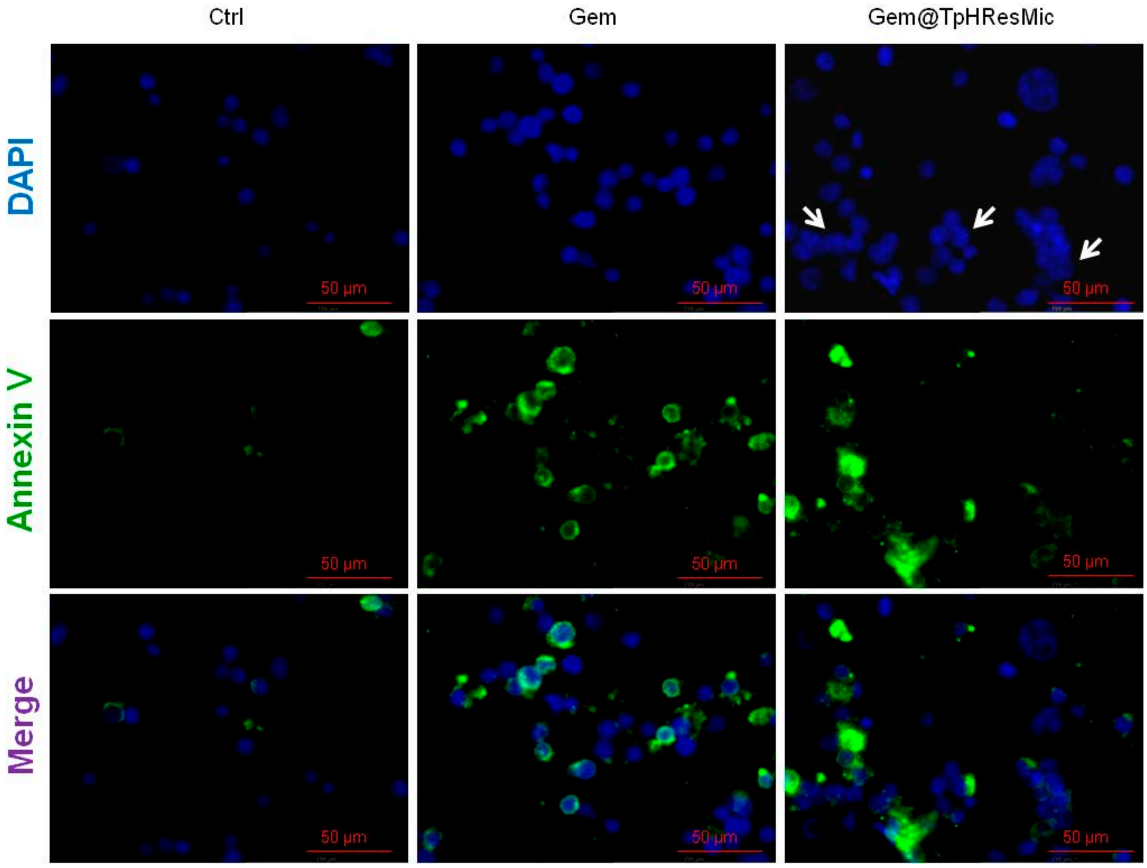

Figure S1. Representative IF images of PANC-1 cell line showing nuclei features (white arrows show polinucleated cells) and Annexin V positive cells (blue: DAPI-stained nuclei, green: Annevin V).

Supplementary S2. Materials and Methods

2.1. Synthesis of copolymers

2.1.1. Synthesis of activated PLGA

A round flask was placed in an anhydrous atmosphere under a constant flow of nitrogen for 20 minutes. 500 mg of PLGA (Resomer 502H) were suspended in 9 mL of dichloromethane (CH_2Cl_2). DCC (11 mg) was added in a molar ratio of 1.5 with PLGA and the solution was stirred for 10 minutes. NHS (6 mg) was added in a molar ratio of 1.5 with PLGA and the solution was stirred for 2 hours. The end of the reaction was indicated by the formation of dicyclohexylurea as by-product. To speed up its formation, the flask was placed in the freezer overnight. The resulting solution was filtered through a Nylon filter

(0.45 μm) to remove the precipitate, re-precipitated with cold diethylether and subsequently dried under reduced pressure.

2.1.2. Synthesis and characterization of PLGA-PEG

330 mg of activated PLGA were dissolved in 5 mL of THF. PEG (141 mg) was added in a molar ratio of 1.5 with PLGA and dissolved in 2 mL of anhydrous DMF. 7 mL of DCM were added to precipitate copolymers and then it was re-precipitated in cold diethylether. The copolymer was purified by centrifugation and the obtained product was dried and collected. The copolymer structure was investigated with FT-IR and ^{1}H-NMR techniques.

2.1.3. Synthesis and characterization of PLGA-PLL

100 mg of activated PLGA were dissolved in 1 mL of DMSO. The resulting solution was added to 4 mL of DMSO solution containing PLL (250 mg) and TEA (2 μL), both in a molar ratio of 1.5 with PLGA. The solution was left under constant stirring overnight. The next day, 1:1 volume of distilled water was added to the solution. The resulting solution was transferred to dialysis membrane (MWCO 3.5 kDa) and dialyzed against distilled water for 2 days. After dialysis, the solution was lyophilized and collected. The copolymer structure was investigated with FT-IR and ^{1}H-NMR techniques.

2.1.4. Synthesis and characterization of PLGA-PLL-DMA

100 mg of PLGA-PLL were solubilized in 2 mL of DMSO. 10 mg of DMA were put into the solution, and then 10 μL of TEA and 10 μL of Pyridine were added. The solution was stirred overnight in a nitrogen chamber. After 24 hours, 10 mL of distilled water at pH = 9 was added to the DMSO solution. The water / DMSO solution was dialyzed in a dialysis bag (MWCO 3.5 kDa) against basic distilled water at pH = 9 for 7 hours, changing the water approximately every hour. The dialyzed solution was freezed-dried for 48 hours. The copolymer structure was investigated with FT-IR and ^{1}H-NMR techniques.

2.1.5. Conjugation of the pegylated-AE105 peptide with PLGA

AE105 peptide conjugated with PEG 5000 was added to the solution containing the activated polymer PLGA in a 1/1.5 molar ratio (PEG-AE105/PLGA). A solution containing 0.05% *v/v* TEA in DMSO (200 uL) was further diluted in DMSO and raised to 1 mL. PEG-AE105 (5 mg) was solubilized in the resulting solution. PLGA copolymer (7.52 mg) was solubilized in 1 mL of DMSO, and the obtained solution was added to the solution containing the pegylated peptide. The resulting solution containing PLGA-PEG-AE105 was left stirring overnight under constant nitrogen flow. The following day, an equal volume of distilled water was added to the solution. The solution was then dialyzed against distilled water in dialysis membrane (MWCO 12-14 kDa) for 2 days. After dialysis, the resulting solution was frozen and lyophilized to obtain the final product. The succesful conjugation was verified with UV-Vis spectroscopy.

2.3.3. Particle size, size distribution and surface charge

After their preparation, the micelles were characterized by means of dynamic light scattering to obtain information about their size, polydispersity index (PDI), and zeta (ζ)-potential, using Malvern Zetasizer Nano instrument (Malvern Ltd., UK). About 1 mL of a 1:50 dilutions of each sample with PBS at two different pH (pH 7.4 and pH 6.8), was pipetted into a disposable polystyrene cuvette (Sarstedt AG & Co., Germany) and the measurements were carried out at 25 ± 0.1 ºC. For the determination of the micelles surface ζ-potential all samples were redispersed in PBS (pH 7.4 and pH 6.8) and each particle suspension (750 μL) pipetted into a disposable folder capillary cell (DTS1070, Malvern, UK).The results are shown as numeric average and standard deviation of the measurements of 3 different samples, each sample measured 3 times.

2.4. Stability studies

In order to estimate the micelles short-term stability, the size distribution of pHResMic and TpHResMic was measured in PBS (pH 7.4). Basically, 200 µL of micelles were incubated in 1.5 mL of physiological media at 37 °C; at defined time points (5, 15, 30, 60, 90, 120, 1440 2880 and 4320 min) a defined volume of sample has been collected, diluted in PBS pH 7.4 in order to analyse the change in size over time. Triplicates of each experiments were performed.

2.5. Evaluation of Drug Encapsulation Efficiency

To calculate the encapsulation efficacy (EE %) values of GEM loaded in micelles, 200 µL of micelles were freeze-dried and after being dissolved with 0.5 mL DMSO was analyzed for drug content by UV–vis spectroscopy (Perkin Elmer Lambda Bio20) exploiting the absorbance peak at 294 nm. GEM concentration was assessed through calibration curve. The EE% values of drug were calculated according the following formula

$$Encapsulation\ Efficacy\ (\%) = \frac{Weight\ of\ drug\ in\ micelles}{Weight\ of\ drug\ added\ initially} \times 100$$

2.6. In vitro drug release study

Studies of Gem release from TpHResMic were performed using Franz cells and experiments were carried out at two different pH (6.8 and 7.4). Briefly, 500 µL of Gem@TpHResMic was placed on the diffusion barrier (area of 0.6 cm^2) constituted by an artificial cellulose acetate membrane (3.5 kDa, Fisher Scientific Milano), which divides donor and receptor cells. Phosphate buffer (PBS, pH 7.4 and 6.8) with 1 % (*w*/*v*) of Tween 80 was selected as receptor medium and it was frequently stirred and retained at a temperature of (37 ± 0.5 °C). In an overall time of 72 h, 0.2 mL was picked up from the receiving compartment at set times, and to provide the sink conditions the equivalent volume of refreshing PBS was included in the receptor cell. The collected fractions were analyzed by UV-Vis to determine the drug content. Each experiment was performed in triplicate and was conducted in three separate Franz cells using three distinct batches of Gem@TpHResMic.

Supplementary S3. Results and discussion

3.1. Characterization of copolymers

The ^{1}H-NMR spectra were consistent with the given structure:

PLGA-PEG ^{1}H-NMR (DMSO d6) *δ: 1.59 (m, [-CH-CH$_3$]); 3.49(s, [–CH$_2$-CH$_2$]); 4.64 (m, [–C(O)-CH$_2$-]); 5.22 (m, [-C(O)-CH-(CH$_3$)–])* (Figure 3A-a).

PLGA-PLL ^{1}H-NMR (DMSO d6) δ: *2.51 (m, [—CH$_2$-(NH$_2$)–]); 3.25 (s, [–CH$_2$-NH–]); 4.20 (m, [–C(O)-CH$_2$-NH$_2$]); 5.12 (m, [–C(O)-CH-(CH$_3$)–])* (Figure 3A-b).

PLGA-PLL-DMA ^{1}H-NMR (DMSO d6) *δ: 1.50 (m, [–C(O)-CH-(CH$_3$)–]); 2.50 (m, [-CH$_3$]); 3.64 (s, [–CH$_2$-CH$_2$–]*) (Figure 3 (a)-c).

The successful conjugations were confirmed by the overlap of the ^{1}H-NMR spectrum of PLGA-PEG, PLGA-PLL and PLGA-PLL-DMA copolymers. In the PLGA-PLL spectrum, the peaks around 3.25 and 2.51 ppm were attributed respectively to the protons of carbon in ε-position of the polylysine side chain, bound (protons q) and unbound (protons p) to the PLGA by means of the NH_2 group, while the Cα proton of the PLL (s) was attributed to the peak around 4.2 ppm. From the ratio of the integrals of the relative peak to the proton (s) of the PLL and of the proton (r) of the PLGA it was determined the % of PLGA linked to the PLL. From the ratio of the integrals of the peak relative to the protons (p) of the PLL, and of the peak relative to the proton (s) of the PLGA, it was possible to determine the % of unconjugated NH_2 groups. This value was used to define the correct molar ratios to be used for the reaction of conjugation of these free NH_2 groups with DMA in the synthesis of copolymer PLGA-PLL-DMA. The conjugation with DMA was confirmed by the

fundamental peak of PLGA-PLL-DMA, about 2.5 ppm, that represented the signal relating to the two methyl groups of the DMA that reacted with the PLL and its value shifted further to the left compared to DMA alone (data not shown).

The FT-IR spectrum of PLGA-PEG (Figure S1 (b)-a) copolymer showed an absorption band at 3504 cm^{-1} corresponding to the terminal hydroxyl groups of poly (ethylene glycol) and glycolic acid, an intense band at 1760 cm^{-1} corresponding to stretching vibration of the carbonyl group (C = O) and the corresponding bands to the stretching of the C-O bond between 1172.4-1061.6 cm^{-1}.

The FT-IR spectrum of PLGA-PLL (Figure S1 (b)-b) copolymer showed a peak at 3330 cm^{-1} corresponding to the terminal amino group of the PLL, and a strong band at 1760 cm^{-1} corresponding to the vibrational stretching of the carbonyl group (C = O).

The overlay of the FT-IR spectrum of PLGA-PLL-DMA (Figure 3B-c) with those of the starting constituents (Figure S1(b) a,b), highlighted the actual conjugation occurred between the PLGA-PEG-PLL and DMA, as it showed the disappearance of the N-H band stretching a 3430 cm^{-1} and the simultaneous appearance of two new bands associated with the stretching of the O-H at 3380 cm^{-1} and the bending at 1440 cm^{-1}. Furthermore, the characteristic peaks of C = O at 1655 cm^{-1} and of C-H at 2940 cm^{-1} were very intense. These were attributed to the carboxyl groups and methyls of DMA. The intensity of the N-H peak at 1540 cm^{-1} resulted stronger due to the generation of amide groups between DMA and PLL.

Finally, the overlapping of UV-Vis spectra of the conjugated copolymer PLGA-PEG-AE105 and of the pristine PEG-AE105 (Figure S (c)), allowed to recognize in the range 260-320 nm, the characteristic absorbance peak of the PEG-AE105 in the copolymer, confirming the successful conjugation reaction between the free COOH group of the PLGA and the terminal NH_2 group of the PEG-AE105 peptide

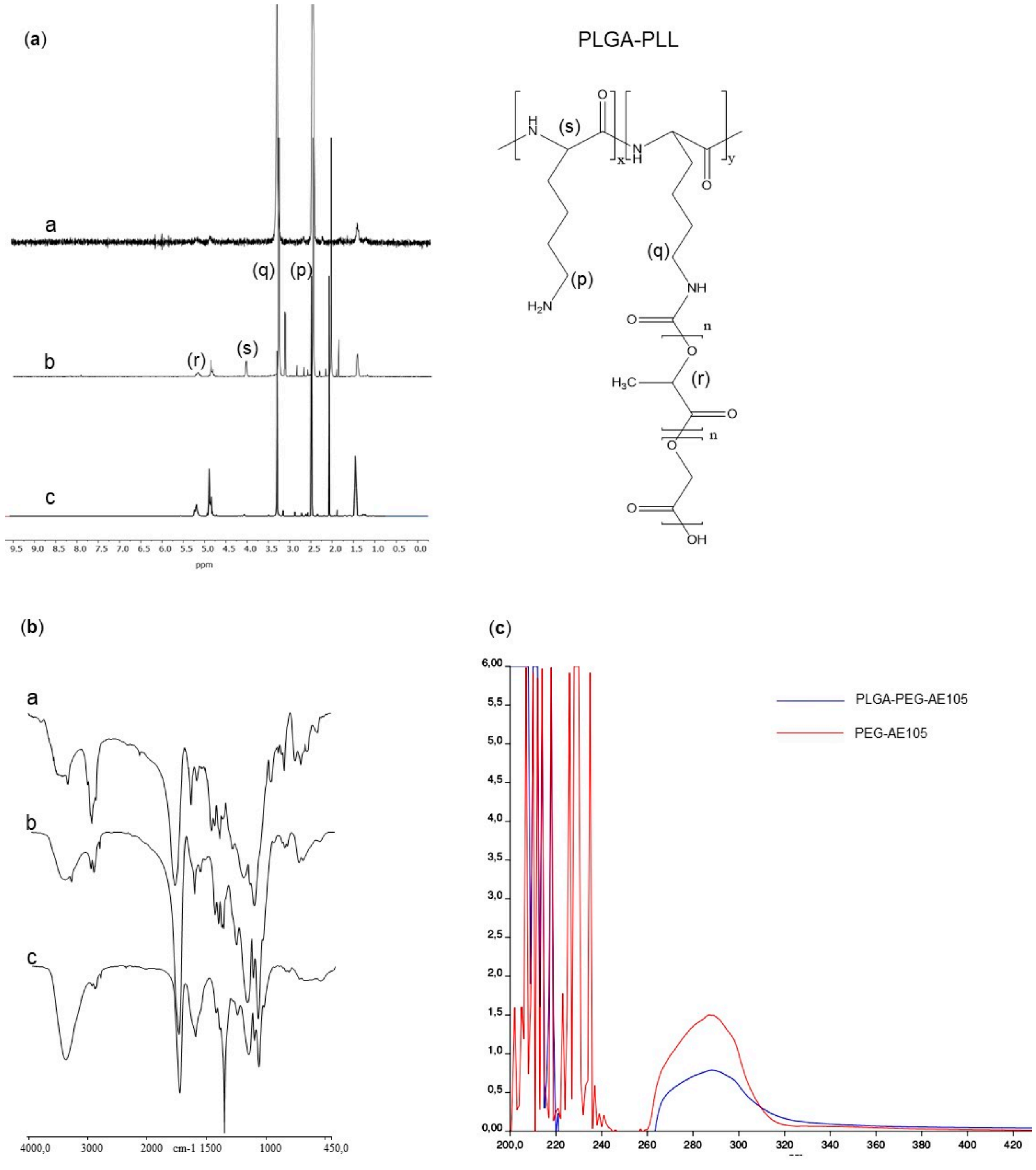

Figure S3. ^{1}H-NMR (**a**) and FT-IR (**b**) spectra of PLGA-PEG a, PLGA-PLL b and PLGA-PLL-DMA c along with the representation of the chemical structure of PLGA-PLL copolymer. UV-Vis Spectra (**c**) of PLGA-PEG-AE105 (blue) and PEG-AE105 (red).
